# Bioorthogonal Chemistry in Cellular Organelles

**DOI:** 10.1007/s41061-023-00446-5

**Published:** 2023-12-16

**Authors:** Veronika Šlachtová, Marek Chovanec, Michal Rahm, Milan Vrabel

**Affiliations:** 1 Department of Bioorganic and Medicinal Chemistry, Institute of Organic Chemistry and Biochemistry of the Czech Academy of Sciences, Flemingovo náměstí 2, 166 10 Prague 6, Czech Republic; 2 University of Chemistry and Technology, Technická 5, 166 28 Prague 6, Czech Republic

**Keywords:** Bioorthogonal reactions, Bioconjugations, Cellular organelles, Click chemistry, Targeting

## Abstract

While bioorthogonal reactions are routinely employed in living cells and organisms, their application within individual organelles remains limited. In this review, we highlight diverse examples of bioorthogonal reactions used to investigate the roles of biomolecules and biological processes as well as advanced imaging techniques within cellular organelles. These innovations hold great promise for therapeutic interventions in personalized medicine and precision therapies. We also address existing challenges related to the selectivity and trafficking of subcellular dynamics. Organelle-targeted bioorthogonal reactions have the potential to significantly advance our understanding of cellular organization and function, provide new pathways for basic research and clinical applications, and shape the direction of cell biology and medical research.

## Introduction

**Fig. 1:**
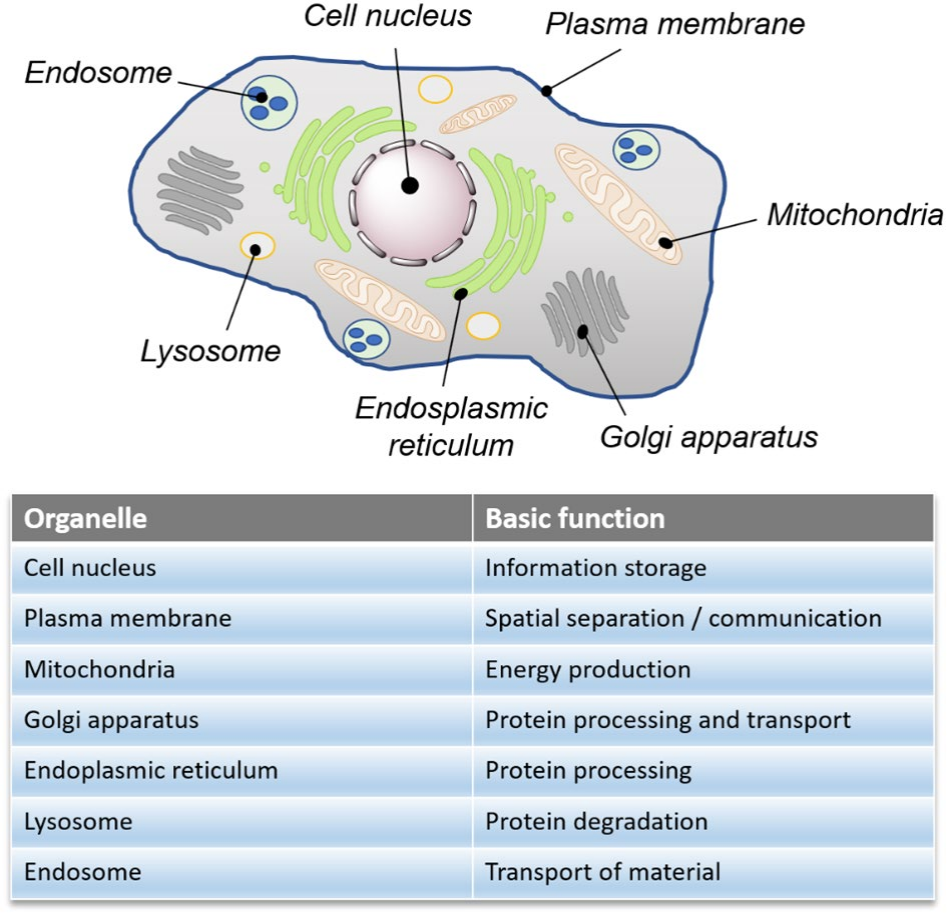
Major cellular organelles and their basic functions

Cellular organelles are the cellular counterparts of organs. These membrane-enclosed vesicles, which contain a wide and diverse range of biomolecules, collectively execute a multitude of functions vital for cells to proliferate and survive [1]. The strategic compartmentalization of cellular processes across diverse compartments provides an evolutionary advantage, allowing cells to adapt more effectively to intrinsic or environmental stress. Equally important are the interconnections that weave the organelles together. The exchange of nutrients and materials, as well as their mutual interactions, form the basis of cellular functions, which play an important role in maintaining our body's harmony. Figure 1 illustrates the major cellular organelles and their respective roles.

Unsurprisingly, dysfunction in cellular organelles has been inextricably linked to a host of pathological conditions [2]. An imbalance in the functioning of cellular organelles or a defect in the way they communicate can often result in disease. For instance, mitochondrial disorders stemming from mutations in mitochondrial DNA (mtDNA) manifest as mitochondrial encephalomyopathies [3]. Neurological diseases have been associated with the endoplasmic reticulum (ER) [4] and lysosomal storage diseases with lysosomes [5]. Predictably, targeting drug molecules to specific cellular organelles is highly relevant from a medical perspective. A growing body of evidence highlights the importance of delivering drug molecules to precise organelles to augment their therapeutic potency [6]. Understanding cellular processes at the subcellular level can provide new insights into biological processes and how they interact with each other. This knowledge can in turn be used to develop innovative therapeutic interventions aimed at preventing or treating disease.

In addition to classical biochemistry and biology approaches [7], modern chemical tools provide an unprecedented means of tracking, labeling, monitoring, and inspecting molecules in individual organelles. Among these tools, bioorthogonal reactions are a particularly valuable addition to the repertoire of chemical transformations that can be performed in living systems without interfering with natural processes or harming the organism. The literature extensively documents the routine application of these biocompatible reactions within live cells [8]. In contrast, there are far fewer examples of reactions performed in an organelle-specific manner. Fortunately, the integration of abiotic chemical reactions within distinct organelles means that cellular processes can now be examined with unparalleled precision.

## Methods for Targeting Cellular Organelles

**Fig. 2:**
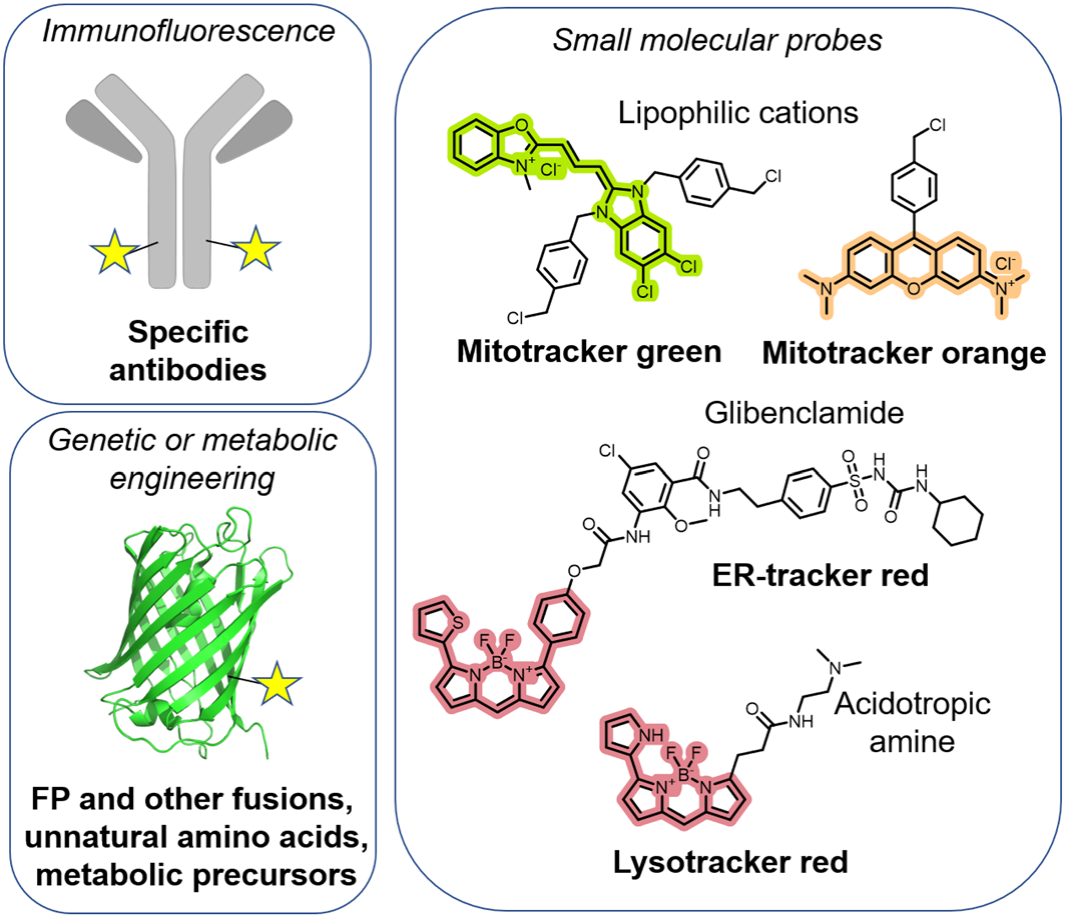
Examples of methods enabling the targeting, visualization, and labeling of cellular organelles

Traditionally, the staining of cellular organelles relies on antibodies that target enzymes or proteins located within specific subcellular compartments [9]. However, this immunofluorescence technique requires the use of fixed and permeabilized cells for antibody-antigen binding to be effective. As a result, the capacity to monitor organelles in their native state in real time is inherently limited by this approach. Alternatively, organelles can be labeled using fluorescent proteins (FPs) [10]. For example, fusing an FP with a signaling peptide facilitates the directed expression of the FP within the designated organelle. Nevertheless, achieving intracellular expression of these modified FPs commonly relies on transfections or transductions, processes that can occasionally prove challenging, particularly when using primary cell lines. Conversely, the employment of small molecular probes offers a straightforward alternative, as the mere addition of a compound to the cells results in its accumulation within the target organelle. Indeed, a wide range of readily available fluorescent molecules can be used to visualize organelles [11]. These include commercial organelle trackers such as mitotrackers, lysotrackers, and ER trackers (Fig. 2). Furthermore, various ingenious molecular designs allow these compounds to be converted into specific stimuli responsive molecular probes reacting to changes or molecules in their environment [12–15]. Similarly, the synergy of genetic manipulation techniques with small molecules is adept at directing diverse probes to distinct cellular compartments [16, 17].

Integrating the bioorthogonality of chemical reactions with organelle targeting is a novel way of investigating and manipulating biological processes at the subcellular level. In this review, we summarize recent examples of bioorthogonal reactions performed within cellular organelles. In delving into these distinct contributions, we examine the type of bioorthogonal reaction used, the method of organelle targeting employed, the information generated, and the problems addressed. The chapters in this review are categorized according to the individual organelles targeted. We have chosen not to include studies that focus on labeling cellular membranes using metabolic glycoengineering, as they have already been recently discussed in a number of excellent reviews [18–21]. Our review also excludes studies involving the utilization of light as a trigger for chemical reactions, which mainly focus on uncaging molecules in the context of cellular organelles [22–25]. Instead, we focus on bioorthogonal reactions driven by alternative forces, such as strains or reagents, as well as organometallic reactions performed in cell organelles. To the best of our knowledge, no comparable review currently exists. We provide a succinct overview of the various advanced bioorthogonal reactions that go beyond conventional cellular applications. The aim of this review is to offer new perspectives on this dynamic subfield of bioorthogonal chemistry and to stimulate interest in state-of-the-art techniques now being used in organic chemistry, tools that can help us unravel the complexity of living systems at the individual organelle level.

### Incorporating Bioorthogonal Groups into Organelles Using HaloTag Technology

**Fig. 3:**
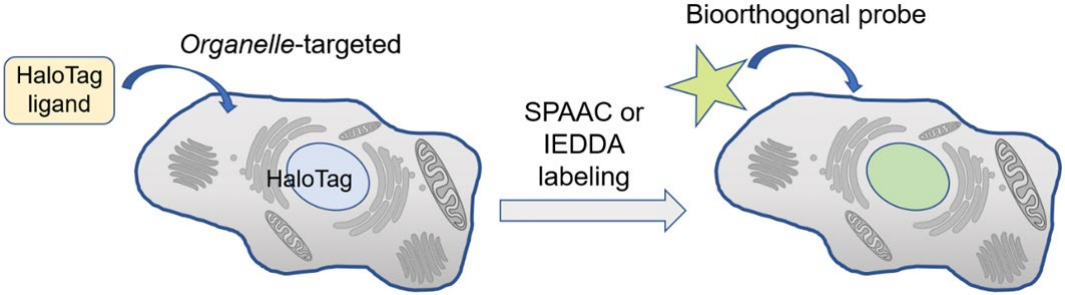
Modification of cellular organelles based on HaloTag technology

There are various strategies for incorporating bioorthogonal functional groups into distinct organelles. One approach involves genetically engineering cellular machinery to selectively introduce these groups into target organelles. For example, Murrey et al. [16] employed HaloTag technology for this purpose [26]. This methodology involves genetically fusing an engineered haloalkane dehalogenase to the protein of interest. Subsequently, upon the addition of a chloroalkane ligand carrying a bioorthogonal group (or another reporter molecule), the ligand covalently attaches to the dehalogenase. As a result, the protein of interest is indirectly labeled with the reporter molecule. Through the formulation of a series of conjugates comprising HaloTag chloroalkane ligands and bioorthogonal reagents, the researchers specifically localized distinct probes within various cellular compartments. They then compared the efficiency of two bioorthogonal reactions within live cells: the strain-promoted azide–alkyne cycloaddition (SPAAC) and the inverse-electron-demand Diels-Alder (IEDDA) reaction (Fig. 3).

Using complementary click molecules, gel electrophoresis, and microscopy analysis, the researchers generated a compelling data set demonstrating differences in the performance of reagents and reactions when conducted inside cells and in distinct compartments. Notably, when dibenzocyclooctyne (DBCO) or bicyclononyne (BCN) ligands were fused to HaloTag in the nucleus, DBCO displayed higher reactivity with the TAMRA azide compared to BCN. Additionally, DBCO exhibited not only accelerated reaction kinetics but also required less reagent to generate a stronger labeling signal. Comparing the IEDDA reactivity of BCN to that of the chloroalkane–*trans*-cyclooctene (TCO) ligand using TAMRA–tetrazine (Tz), the difference between the two reactions inside the cells was rather small. Intriguingly, in the context of live cells, the efficiency of the DBCO–azide bioorthogonal pair was comparable to the notably faster TCO–Tz reaction. In addition, it was found that using DBCO or BCN as labels and azide during the labeling step yielded significantly better results than the reverse pairing of bioorthogonal groups.

Labeling intracellular targets within live cells is a formidable challenge, demanding bioorthogonal reagents that are not only capable of efficiently penetrating cell membranes but also resilient to the cellular environment. In this context, assessing reaction kinetics solely in vitro is insufficient for determining the feasibility and efficiency of the reaction. The authors therefore emphasized the importance of cell permeability, stability, and side reactivity of bioorthogonal reagents in intracellular labeling. For instance, strained alkynes generated higher background signals compared to TCO–Tz labeling, despite H–Tz producing higher background signals at higher concentrations. Notably, the use of more reactive TCO derivatives such as sTCO [27] and dTCO [28] yielded encouraging results for efficiency, selectivity, and labeling speed.

In summary, this study aptly illustrates the importance of considering a variety of factors when selecting the optimal bioorthogonal labeling pair for a particular application. Moreover, it introduces a practical method for precisely guiding chemical reagents to specific locations within live cells. When combined with other self-labeling tag technologies, this strategy has the potential to expand its scope, enabling the targeting of two or more reagents to specific subcellular compartments [29]. On the other hand, the fusion of a relatively large HaloTag protein (33 kDa) can affect the distribution of the target protein or even its structure and function. These disadvantages must be carefully considered when using the method.

### Incorporating Bioorthogonal Groups into Organelles Using Genetic Code Expansion

**Fig. 4:**
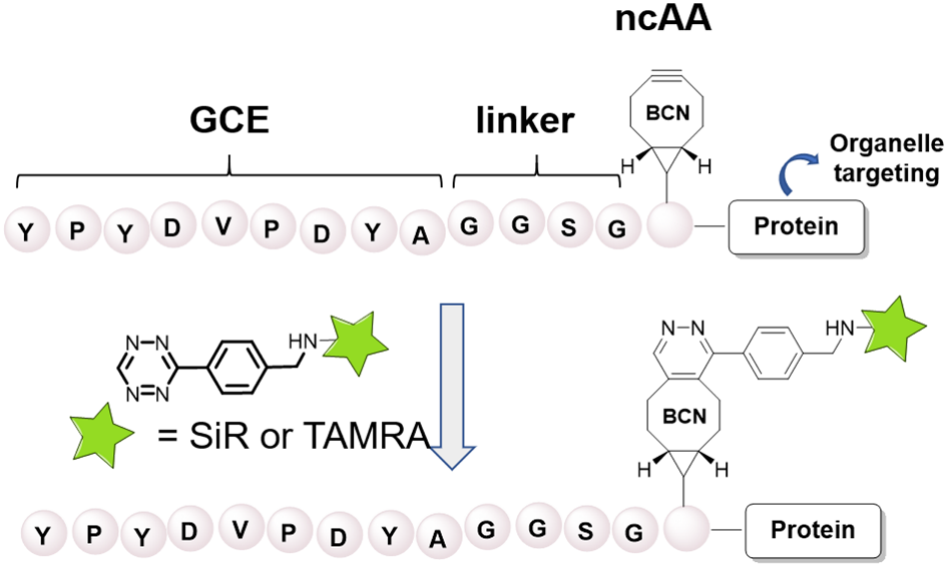
Genetic code extension technique enabling the incorporation of BCN-containing amino acids into proteins across various cellular organelles

The genetic incorporation of noncanonical amino acids (ncAAs) is a powerful technique for inserting bioorthogonal reactive groups into proteins [30–33]. These groups can be used for subsequent click labeling and visualization. In principle, ncAAs can be introduced anywhere within the protein sequence in response to an in-frame amber stop codon. However, the practical identification of suitable labeling sites can be a demanding and time-intensive task, primarily because of two key factors. First, the placement of the amino acid within the sequence must not disrupt the structure or function of the protein. Second, the efficiency of the incorporation can be variable and influenced by both the local environment and sequence context [34]. To overcome these challenges, Segal et al. developed a genetic code expansion (GCE) method based on a short, N-terminal 14 amino acid tag (YPYDVPDYAGGSGX), called the GCE tag. This tag can be fused to diverse proteins through a short glycine–glycine–serine–glycine (GGSG) linker, facilitating the incorporation of a BCN-containing amino acid via an orthogonal tRNA–tRNA synthetase pair (Fig. 4) [17].

Having established expression within live mammalian cells, the authors demonstrated the successful labeling of various organelles. This was achieved by fusing the GCE tag to proteins known to reside in specific subcellular locations, including the plasma membrane (GFP-CAAX), peroxisomes (GFP-SKL), lysosomes (Lamp1), multivesicular bodies (CD63), the ER (ER^cb5^TM), exosomes (Exo70), and mitochondria (Mito^cb5^TM or mito-DsRed). Interestingly, the labeling probe also played an important role. Specifically, when the SiR-Tz probe was used, the labeling of multivesicular bodies, ER, mitochondria, and exosomes exhibited nonspecific signals. However, substituting this probe with the structurally similar TAMRA-Tz led to specific staining in all cases except for mitochondria. This study demonstrates that, in addition to targeting efficiency and specificity, the selection of labeling probe can also substantially impact experimental outcomes. Therefore, to achieve the ideal results, it is crucial to carefully consider and optimize each parameter.

### Targeting Organelles with Small Molecular Probes

Targeting organelles using small molecules is one of the most widely employed strategies. Compared to the previous examples above, this approach boasts several notable advantages, including the ability to specifically target individual organelles without the need to genetically manipulate the cell or organism. Typically, introducing the probe is sufficient to achieve selectivity. The choice of molecular probes varies depending on the specific organelle and intended application. In principle, metabolic precursors that utilize the biosynthetic machinery of cells can be used [35], as can probes that do not require incorporation by endogenous enzymes. Detailed descriptions of the different probe types are provided in subsequent sections of this review, each focusing on the targeting of distinct organelles.

## Reactions in the Cell Nucleus

The use of organelle-specific bioorthogonal probes to label and visualize nucleic acids plays a pivotal role in elucidating cellular processes and the flow of genetic information. Leveraging the power of metabolic labeling, scientists have deployed modified nucleosides and nucleotides to selectively tag DNA within the nucleus. These modified nucleic acids, when labeled with fluorescent or fluorogenic bioorthogonal probes, emit light within the nucleus. This allows researchers to visualize nuclear dynamics and analyze nuclear architecture and organization with exceptional precision and sensitivity. Other notable tools include DNA–DNA interstrand cross-links (ICLs), sensitizers for photodynamic therapy, and DNA–protein interactions. The following paragraphs summarize these methodologies and highlight their principles, applications, and contributions to our understanding of cellular biology and nuclear processes.

### Metabolic Labeling with Nucleosides

**Scheme 1:**
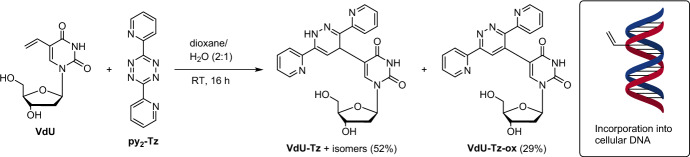
Reaction between VdU and 3,6-di(2-pyridyl)-1,2,4,5-tetrazine (py_2_-Tz) in the presence of ambient oxygen

The traditional approach to metabolic labeling and detection of cellular DNA involves the use of 5-bromo-2′-deoxyuridine (BrdU), a thymidine analog typically detected by immunohistochemical staining [36]. However, BrdU detection is hampered by the limited ability of antibodies to penetrate tissue. In this context, bioorthogonal chemistry has proved to be a more effective approach [37]. Perhaps the most widely employed metabolic DNA label, ethynyl-2′-deoxyuridine (EdU), can be sensitively detected through a chemical reaction called Cu(I)-mediated azide–alkyne cycloaddition (CuAAC) [38]. Additionally, a range of clickable nucleosides, such as F-*ara*-EdU, have been developed to mitigate the toxicity of EdU [36]. However, there is a continued need for the development of alternative bioorthogonal ligations in DNA imaging, especially in mutually orthogonal experiments [38]. In response to this demand, Luedtke and colleagues [39] demonstrated that the modified nucleoside 5-vinyl-2′-deoxyuridine (VdU) can be metabolically incorporated into cellular DNA. The VdU-labeled DNA can then be detected through an IEDDA ligation with a fluorescent tetrazine, operating with a second-order rate constant *k* ≈ 0.02 M^−1^ s^−1^ (Scheme 1).

The ability of endogenous enzymes to incorporate VdU into DNA was assessed in HeLa, U2OS, A549, Vero, and MRC-5 cells, employing doses ranging from 1 to 100 µM VdU over 16 h, followed by cell fixation and TAMRA–Tz labeling. Upon the addition of aphidicolin, an inhibitor of DNA synthesis, VdU labeling was no longer observed, underscoring the selective metabolic integration of VdU into DNA.

The mutual orthogonality of VdU and EdU incorporation was evaluated by sequential treatment of HeLa cells. The cells were initially treated with a pulse of EdU and subsequently with a VdU “chase.” This was followed by fixation and staining with TAMRA–Tz and Alexa Fluor azide, respectively. Quantitative image analysis demonstrated comparable efficiencies for both VdU and EdU incorporation and labeling.

This orthogonal chemical labeling framework was further expanded by including the immunohistochemical staining of BrdU. The compatibility of the methods was investigated by sequential treatment of HeLa cells using BrdU (30 µM), VdU (30 µM), and F-*ara*-EdU (30 µM) [36]. Subsequent staining revealed well-resolved, non-overlapping fluorescent signals with distribution characteristic of an S-phase [40]. Importantly, the order in which the nucleosides were added did not influence these patterns, which suggests that the metabolic integration and detection of these labels are mutually compatible.

**Scheme 2:**
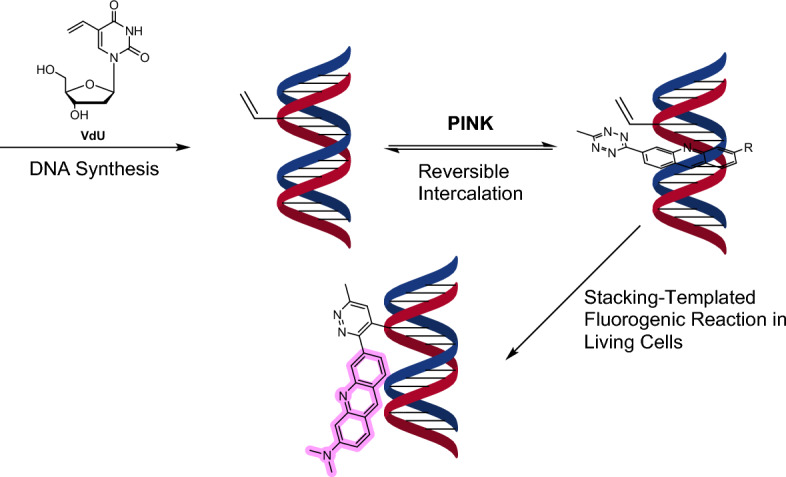
Vinyl-modified cellular DNAs are generated by the incorporation of VdU into living cells and visualized using the PINK probe

In a subsequent study, the same group devised a dual amplification strategy for nucleic acid-templated reactions. They used a fluorogenic intercalating agent that participates in an IEDDA reaction with DNA or RNA containing VdU or 5-vinyl-uridine (VU), respectively [41]. The study revealed the reversible and high-affinity intercalation property of a novel acridine–tetrazine molecule called PINK (*K*_D_ = 5 ± 1 µM), which increased the cycloaddition reaction rate on duplex DNA by an impressive 60,000-fold (590 M^−1^ s^−1^ vs 0.01 M^−1^ s^−1^ for the untemplated reaction). Additionally, this transformation is fluorogenic in nature, facilitating nucleic acid detection within live cells under no-wash conditions. PINK combines a fluorescent intercalator [42] with a tetrazine serving as both a bioorthogonal moiety and a fluorescence quencher (Scheme 2) [43]. Notably, PINK enabled the labeling of native chromatin within live HeLa cells. Excellent colocalization was observed between PINK and the noncovalent nuclear stain Hoechst 33342. At low micromolar concentrations, PINK also proved nontoxic. Notably, this approach was effectively extended to vinyl-modified RNA in HeLa cells, where 5-vinyl uridine was used as the metabolic precursor.

Metabolic VdU incorporation and bioorthogonal labeling were elegantly combined by Linden et al. They developed an iodide-substituted BODIPY–tetrazine probe, which becomes an efficient photosensitizer only after the IEDDA reaction [44]. Using this approach, the authors achieved the nucleus-specific generation of singlet oxygen, leading to cancer cell death after irradiation. This work is a good example of how the subcellular specificity of chemical reactions can be harnessed for therapeutic purposes, particularly in the context of photodynamic therapy.

**Fig. 5:**
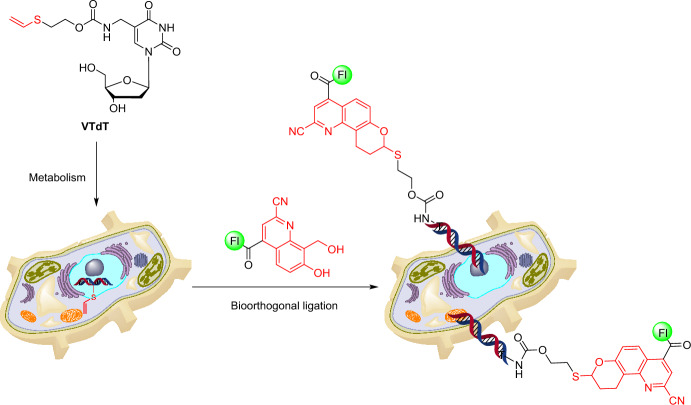
VTdT metabolic incorporation and cellular DNA labeling after bioorthogonal ligation with fluorescein-labeled *o*QQF

Broadening the range of both in vitro and in vivo DNA labeling application, Tang et al. introduced a novel DNA labeling strategy based on a catalyst-free bioorthogonal ligation involving vinyl thioether-modified thymidine (VTdT) and *ortho*-quinolinone quinone methide (*o*QQM) [45]. Using the newly designed VTdT, labeling tags were metabolically introduced into cellular DNA and then conjugated with fluorophores within the cellular environment (Fig. 5).

The ligation of *o*QQF with single-stranded DNA (ssDNA) containing VTdT was completed within 6 h at a rate constant of 2.80 ± 0.2 × 10^−2^ M^−1^ s^−1^. In a similar reaction with double-stranded DNA (dsDNA), the ligation finished within 1 h at a rate constant of 0.42 ± 0.07 M^−1^ s^−1^. VTdT was found to be nontoxic and well tolerated by HeLa cells up to a concentration of 1000 µM over a 24-h period. Notably, VTdT was successfully used to metabolically incorporate labels into cell nuclei as well as mitochondrial DNA. This was validated by colocalizing signals with Hoechst nuclear staining dye and MitoTracker Deep Red mitochondrial dye. Note that in this study the labeling reaction with *o*QQF was carried out on fixed cells.

### Metabolic Labeling with Nucleotides

**Fig. 6:**
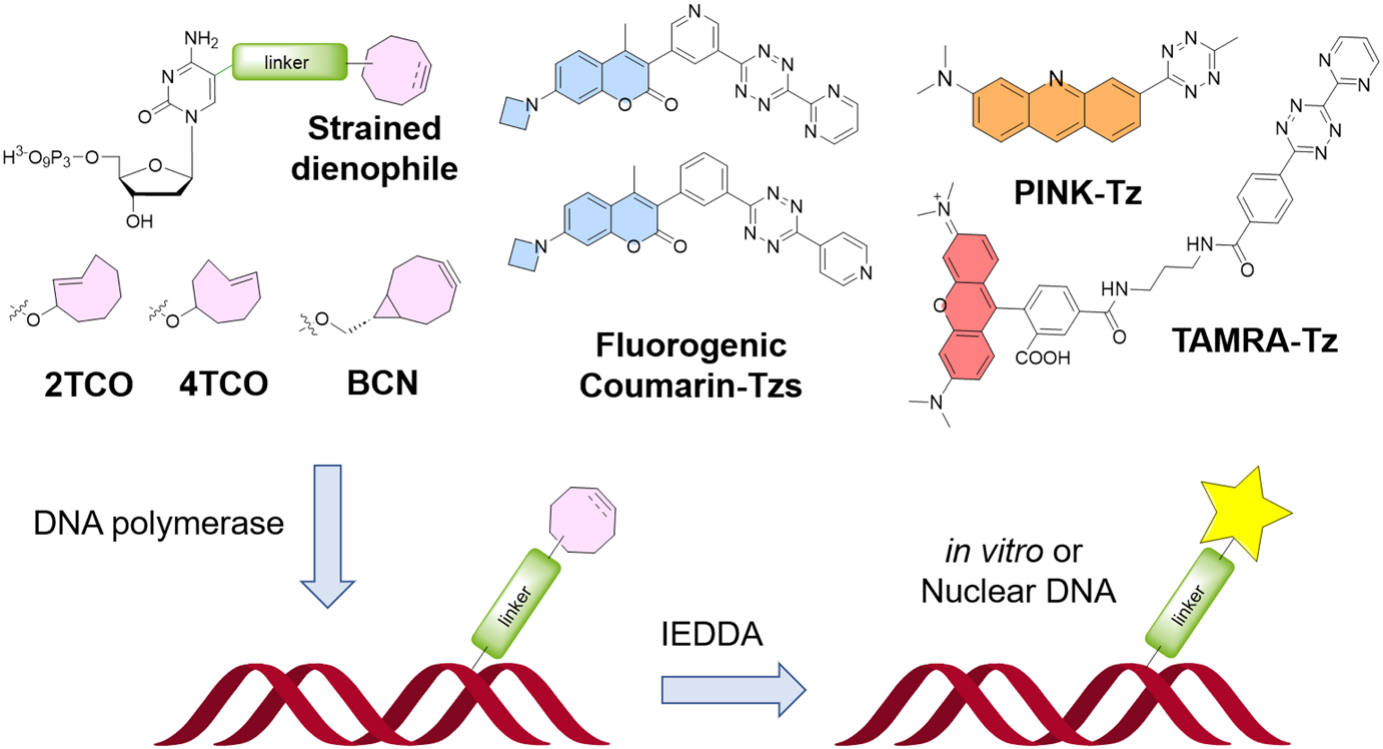
Metabolic labeling of nuclear DNA with triphosphates bearing different bioorthogonal groups

Adopting an alternative approach to nuclear DNA labeling, Hocek and colleagues prepared a series of TCO- and BCN-modified nucleoside triphosphates [46], which were first evaluated in vitro and then efficiently introduced into living cells using SNTT1, an artificial molecular transporter system previously developed by the group [47]. For successful transport, nuclear integration, and subsequent labeling of the modified probes within the cells, an extended PEG3 linker was required. Within the cellular context, various tetrazine conjugates were assessed for DNA labeling, including fluorogenic coumarins [48], PINK tetrazine [41], and the commercially available TAMRA–Tz (Fig. 6).

Interestingly, distinct reaction dynamics were observed between individual reaction partners. One of the fluorogenic coumarin tetrazines effectively labeled all PEG3-linked dienophiles within cells. PINK exhibited superior performance when paired with BCN, while the commercial TAMRA–Tz proved effective in fixed cells. The latter derivative displayed exceptional contrast based on cell cycle analysis by flow cytometry, allowing cells to be quantified at various stages of the cell cycle.

Following a similar strategy, Meier and colleagues developed a lipophilic prodrug incorporating a 2TCO-containing cytidine triphosphate [49], which can diffuse across cell membranes [50]. Subsequently, the researchers explored a range of fluorogenic tetrazine dyes for the purpose of labeling both cellular and viral DNA. In line with the results of Hocek et al. [46], a precisely optimized fluorogenic coumarin proved the optimal choice for intracellular DNA labeling within live cells.

### Intercalation-Enhanced Click Reaction on Nuclear DNA

**Scheme 3:**
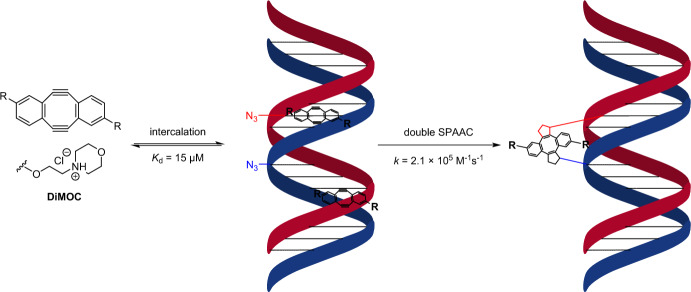
Two-step process involving noncovalent DiMOC intercalation followed by a click reaction to form an ICL between opposing strands of azide-containing DNA

DNA–DNA ICLs are highly toxic products of bifunctional electrophiles, such as phosphoramide mustard or cisplatin [51]. Their toxicity, which is mainly caused by obstructing transcription and replication processes [52], makes them both efficient chemical weapons and chemotherapeutic agents [53]. In vivo-applicable approaches for the chemoselective preparation of DNA–DNA ICLs are scarce. In response to this need, Luedtke et al. [54] designed a two-step method for ICL formation based on dibenzocycloocta-1,5-diyne (“CODY”) [55], also known as Sondheimer diyne [56]. To improve the poor water solubility of the compound, the group introduced two morpholino side chains to CODY, rendering it partially protonated under physiological conditions. This alteration yielded the DiMOC analog, which exhibited an affinity for calf thymus DNA with a *K*_d_ value of 15 ± 7 µM. The capacity of DiMOC to generate DNA–DNA ICLs was investigated through its interaction with azide-containing duplex DNA, prepared by enzymatic extension in the presence of 5-(azidomethyl)-2′-deoxyuridine (AmdU) triphosphate [57]. The ensuing reaction proceeded efficiently and in line with the close proximity of the bioorthogonal groups. This led to the formation of well-defined DNA–DiMOC–DNA ICL products with exceptionally fast kinetics (*k*_app_ = 2.1 ± 0.2 × 10^5^ M^−1^ s^−1^), specifically for the SPAAC reaction. The impressive 21,000-fold rate enhancement observed was attributed to the proximity effect (Scheme 3).

The cytotoxicity of the combination of DiMOC and pivaloyl-protected AmdU was then assessed within living cells. In this context, HeLa cells remained structurally intact even in the presence of up to 30 µM of each individual reagent over a span of 72 h. However, the more delicate acute myeloid leukemia cell lines displayed tolerance to lower doses (3 and 10 µM). Notably, when both probes were applied simultaneously, a cell type-specific response developed, yielding a synergistic effect. Specifically, AmdU–DiMOC–AmdU ICL adducts were exclusively detected in samples jointly treated with POM–AmdU and DiMOC. The purified DNA was then inspected for the presence of DNA–DNA ICLs using denaturation–renaturation analysis [58] and ultrahigh performance liquid chromatography–mass spectrometry (UPLC–MS). This work adeptly underscores how the prearrangement of reagents can lead to improvements in reaction kinetics.

### Fluorogenic Visualization of DNA–Protein Interactions

**Fig. 7:**
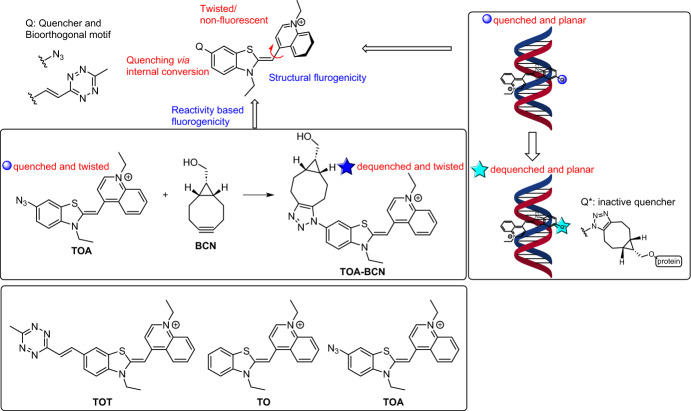
Proposed AND-type double fluorogenicity of bioorthogonally functionalized, environmentally sensitive probes. As a representative example of these DNA sensors, TOA exhibits reactivity (quenched and dequenched states) and structure-based fluorogenicities (twisted and planar conformations). Structures of selected TO derivatives are also shown

The development of methods for tracing DNA–protein interactions in vivo is an important aspect of chemical biology research [59]. Numerous methods have been developed for investigating interactions between nucleic acids and proteins. These methods include chromatin immunoprecipitation (ChIP) [59], electrophoretic mobility shift assays (EMSAs) [60], pull-down assays [61], as well as techniques that use fluorescent detection with intercalating dyes like thiazole orange (TO) or reporter assays that use nucleosides primed to sense DNA–protein interactions [62]. While these methods are adept at detecting DNA–protein interactions in vitro, there is a scarcity of techniques for visualizing these interactions within live cells. These limitations inspired Kele and co-workers to design and develop bioorthogonal probes based on the intercalating dye TO. When a bioorthogonal reaction occurs simultaneously with a DNA interaction, these probes become highly fluorescent [63]. Specifically, the incorporation of thiazole orange azide (TOA) and thiazole orange tetrazine (TOT) moieties into the TO framework results in remarkable fluorogenicity (Fig. 7). The complete fluorescence of these double-fluorogenic probes can be restored only when they simultaneously interact with DNA and undergo covalent conjugation to a site-specifically modified protein of interest (POI).

As expected, the initial experiments validated the double-fluorogenic characteristics of TOA. The fluorogenic behavior of the probes was evaluated both in the presence and absence of BCN and DNA, highlighting a dual fluorogenic response. Notably, TOT emerged as the most suitable probe for cellular labeling due to its rapid kinetics in ligation with BCN, completing the reaction in just 2 h.

First, unlike its cytotoxic TO counterpart, TOT exhibited no adverse impact on cell viability, possibly due to the structural modification with the tetrazine scaffold. Second, the ability of TOT to selectively detect cellular protein–DNA interactions was explored using the model POI candidates lamin A [64] and H2B [65], two proteins that are pivotal in chromatin organization and transcription regulation. To achieve this, the C-termini of these POIs were fused with HaloTag, a self-labeling enzyme tag. The modified cells carrying these POI–HaloTag constructs were exposed to HaloBCN. This resulted in the bioorthogonal adaptation of the modified proteins, which were then treated with TOT. Lastly, confocal microscopy analysis carried out on washed and fixed cells confirmed the membrane permeability of TOT and its effectiveness in detecting protein–DNA interactions.

## Reactions in Lysosomes

Lysosomes are acidic vesicles involved in numerous cellular functions such as cell homeostasis and immunity [66]. Selective labeling of lysosomes, which are essential for cellular waste disposal and various metabolic processes, presents a unique challenge due to their complex and dynamic nature. To address this challenge, the application of bioorthogonal reactions has attracted significant attention, providing a novel means of labeling and visualizing lysosomes with high specificity and minimal interference with cellular function. In this section, we discuss the different methodologies that have used bioorthogonal chemistry for lysosomal labeling, highlighting their principles, advantages, and contributions to our understanding of lysosomal biology and its role in cellular processes.

### Tracking of Stressed Lysosomes via pH-Responsive Probes

**Fig. 8:**
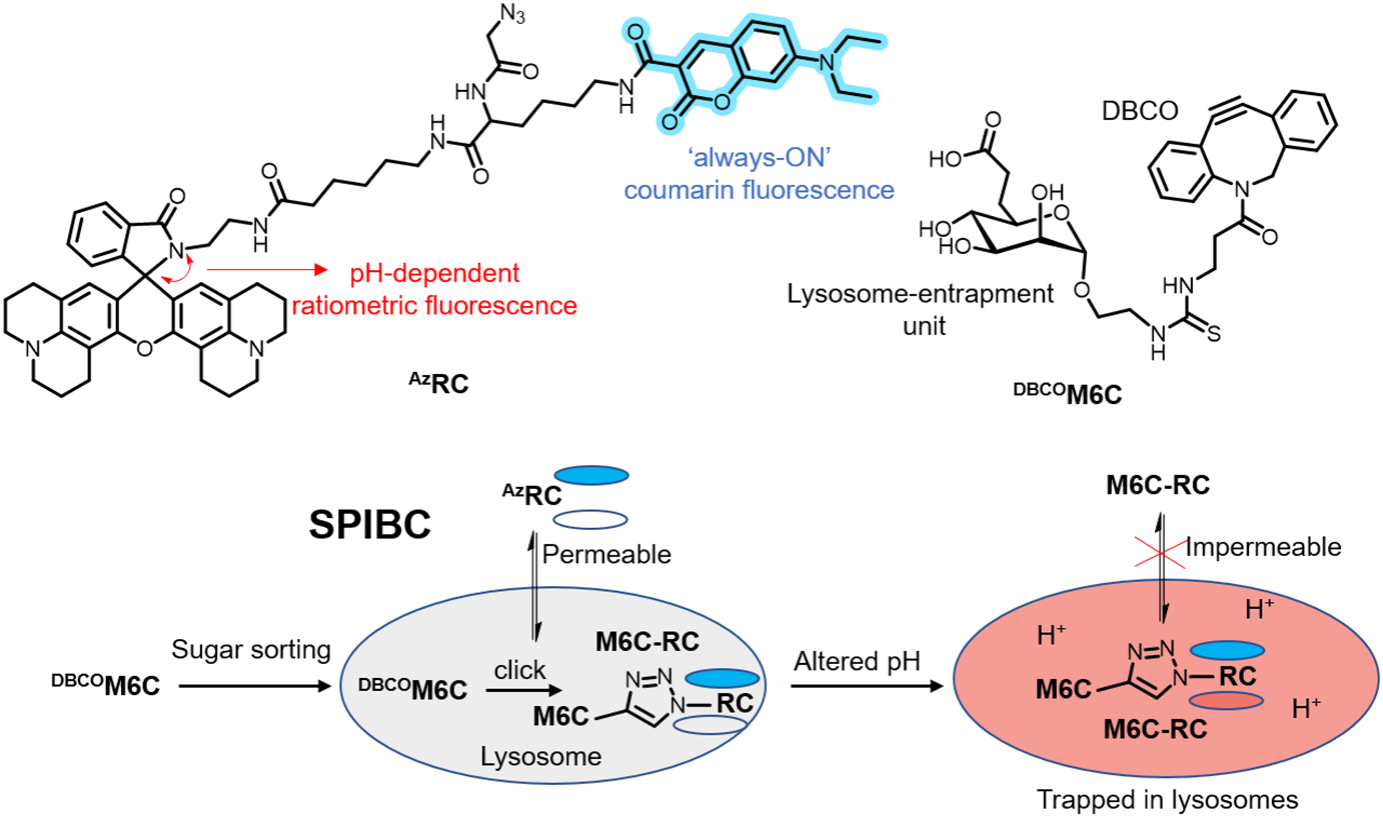
Formation of ratiometric, pH-dependent, lysosome-entrapped M6C-RC probe in live cells

Lysosomal targeting is commonly performed using various probes that become protonated in the acidic environment of lysosomes. This leads to the sequestration and entrapment of the probes within the organelle [67]. However, the drawback of these acidotropic sensors is that they tend to disperse from lysosomes as pH levels increase. Consequently, these probes cannot be used to study abnormal and stressed lysosomes, which often exhibit higher pH values. Overcoming this limitation, Han and co-workers developed a sugar-sorting-pathway-directed intraorganelle bioorthogonal conjugation (SPIBC), which allows lysosomes to be targeted independently of pH (Fig. 8) [68].

For this method, a dibenzocyclooctyne (DBCO)-appended mannose 6-carboxylate (^DBCO^M6C) is actively transported into lysosomes through an endogenous mannose 6-phosphate (M6P) sorting pathway. Inside the lysosomes, ^DBCO^M6C then undergoes a SPAAC reaction with a diffusible azide-functionalized rhodamine-lactam/coumarin construct (^Az^RC), resulting in the formation of M6C-RC within the lysosomes. Once formed within the organelle, M6C-RC remains trapped within the lysosome regardless of the acidity level. Importantly, M6C-RC exhibits pH-sensitive ratiometric fluorescence based on the closing and opening of the rhodamine lactam ring in response to variations in lysosomal pH. The researchers applied the methodology to intralysosomal reaction experiments. They observed a greater increase in lysosomal pH in necrosed cells compared to necrosing cells, whereas lysosomal pH changes remained similar in apoptosed cells compared to cells undergoing apoptosis. Therefore, the method proved adept at tracking stressed lysosomes and distinguishing between necroptosis and apoptosis based on lysosomal pH alterations.

**Fig. 9:**
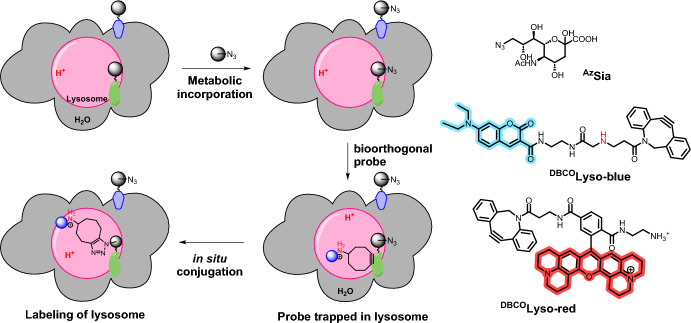
OMGL for covalent lysosomal tagging. OMGL is achieved via metabolic incorporation of ^Az^Sia into cellular glycoproteins and acidity-driven lysosomal accumulation of ^DBCO^Lyso-Blue, leading to an organelle-specific bioorthogonal reaction

Subsequently, the same research group employed a similar approach to target lysosomes [69]. In this instance, the team devised acidotropic agents ^DBCO^Lyso-Blue and ^DBCO^Lyso-Red, containing a lysosomal-targeting amine linker. ^DBCO^Lyso-Blue featured the “always-on” fluorescent coumarin while ^DBCO^Lyso-Red incorporated rhodamine X–lactam (ROX–lactam) with red fluorescence triggered by acidity changes (Fig. 9). Metabolic glycan labeling (MGL) was employed as a strategy to achieve efficient lysosomal retention. Specifically, an organelle-targeted variant of MGL (OMGL) was implemented, involving the incorporation of 9-azidosialic acid (^Az^Sia) into lysosomal membrane proteins.

To establish the effectiveness of the probes, their reactivity with ^Az^Sia was first verified. Lysosomal targeting in HeLa cells was then validated through colocalization studies with GFP-tagged lysosome-associated membrane protein 1 (GFP-Lamp1). Subsequently, the team successfully confirmed acidity-mediated accumulation of the probe by introducing bafilomycin A1 (Baf-A1), a potent V-ATPase inhibitor that neutralizes lysosomal acidity.

The researchers successfully combined the developed OMGL with canonical MGL, allowing them to simultaneously label both the cell surface and lysosomal membranes using different fluorophores. The application was then extended to visualize permeabilized lysosomes. This technique is used to investigate lysosomal membrane permeabilization (LMP), a phenomenon often associated with various cellular death pathways. To induce LMP, the hydrochloride l-leucyl-l-leucine methyl ester (LLOMe) was used in combination with FITC-labeled dextran, a marker that can escape from LMP-positive lysosomes. OMGL demonstrated its ability to track lysosomes undergoing LMP. The versatility of this labeling technique in studying lysosomes across diverse cell death routes was showcased using different cell lines, including Raw 264.7 and HT108.

**Fig. 10:**
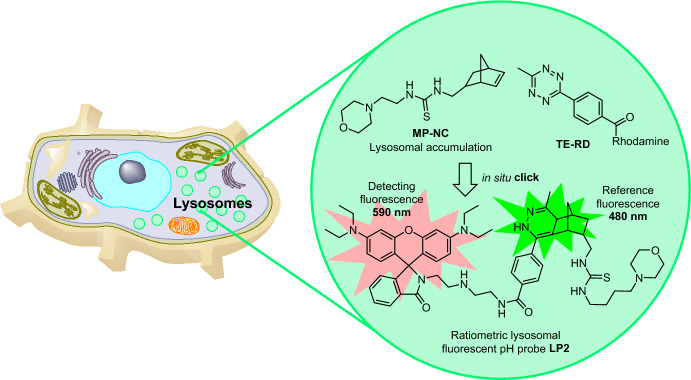
Intralysosomal IEDDA combining fluorogenic reaction between tetrazine and norbornene with ratiometric, pH-dependent fluorescence of a rhodamine dye

Employing a similar strategy, Feng et al. leveraged the ratiometric fluorescence properties of specific rhodamine dyes, in this case coupled with the bioorthogonal IEDDA reaction [70]. The authors opted to exploit the fluorogenic attributes of IEDDA in designing the ratiometric pH-responsive probe LP2. This approach juxtaposed the green fluorescence of the formed dihydropyridazine click product [71] as a reference signal against the red fluorescence of rhodamine for detection. LP2 was designed to form in situ within lysosomes through the IEDDA reaction. The probe consists of a norbornene derivative containing a morpholine moiety (MP–NC) to ensure localization within the lysosomes and a tetrazine-functionalized rhodamine dye (TE–RD) as the detecting fluorophore, selected for its pH-dependent fluorescence (Fig. 10). The feasibility of the in situ click product formation was assessed using the MCF-7 cell line. When the cells were sequentially treated with MP–NC and then TE–RD, distinct green fluorescence emerged in the cells within 10 min. The localization of this fluorescence was confirmed using a commercial lysotracker, indicating that TE-RD entered lysosomes and underwent a reaction with MP-NC to create the LP2 probe within the organelle. The dual fluorescence attributes of the LP2 probe enable precise measurement and monitoring of even subtle pH changes, making it a valuable tool for studying dynamic fluctuations.

### Formation of Phosphorescent Probes in Lysosomes

**Fig. 11:**
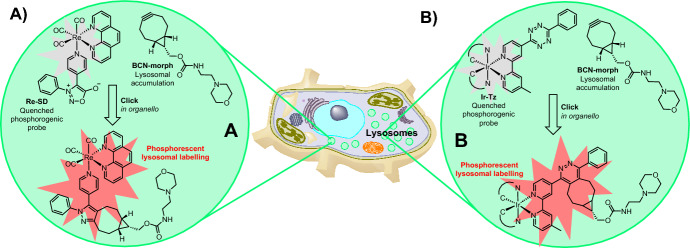
Phosphorogenic labeling of lysosomes achieved using **a** the strain-promoted sydnone–alkyne cycloaddition (SPSAC) reaction and **b** the IEDDA reaction

Fluorophores commonly employed in bioimaging studies are not without their limitations. As an attractive alternative, phosphorescence, with its long lifetime, allows biological processes to be visualized and monitored with a high degree of accuracy [72]. Furthermore, phosphorogenic probes boast the advantage of being able to generate phosphorescent products from two initially nonemissive starting materials, leading to better signal-to-noise ratios [73]. In this context, Shum and co-workers developed rhenium(I) polypyridine complexes [74] coupled to sydnones as bioorthogonal functional handles [75]. Under irradiation, these complexes displayed weak luminescence, possibly due to the polar nature of the sydnone ring and its π-conjugation between the sydnone unit and the pyridine moiety. However, when combined with the strained alkyne BCN-OH, the rhenium(I) complexes formed pyrazole products, which exhibited much stronger emission, highlighting the phosphorogenic properties of the sydnones (Fig. 11A).

The cytotoxicity of the rhenium(I) complexes in HeLa cells was found to be suitable for live cell imaging. Employing a strategy involving a BCN–morpholine (BCN–morph) derivative to target lysosomes, the researchers observed compelling staining and notably enhanced emission in BCN–morph treated cells incubated with the Re–SD complex.

In a subsequent investigation, the same research team harnessed tetrazines as the quenching moiety in conjunction with iridium(III) polypyridine complexes known for their extended excited-state lifetimes (Fig. 11B) [76]. Intriguingly, the click product arising from the reaction of the iridium–tetrazine probe with TCO-OH did not exhibit a notable enhancement in phosphorescence, potentially attributed to the quenching characteristics of the dihydropyridazine unit [77]. In contrast, the pyridazine click product obtained from the reaction of the probe with BCN–OH showed a substantial increase in phosphorescence, enabling effective imaging of lysosomes in cells.

### Click-to-Release Reaction in Lysosomes

**Fig. 12:**
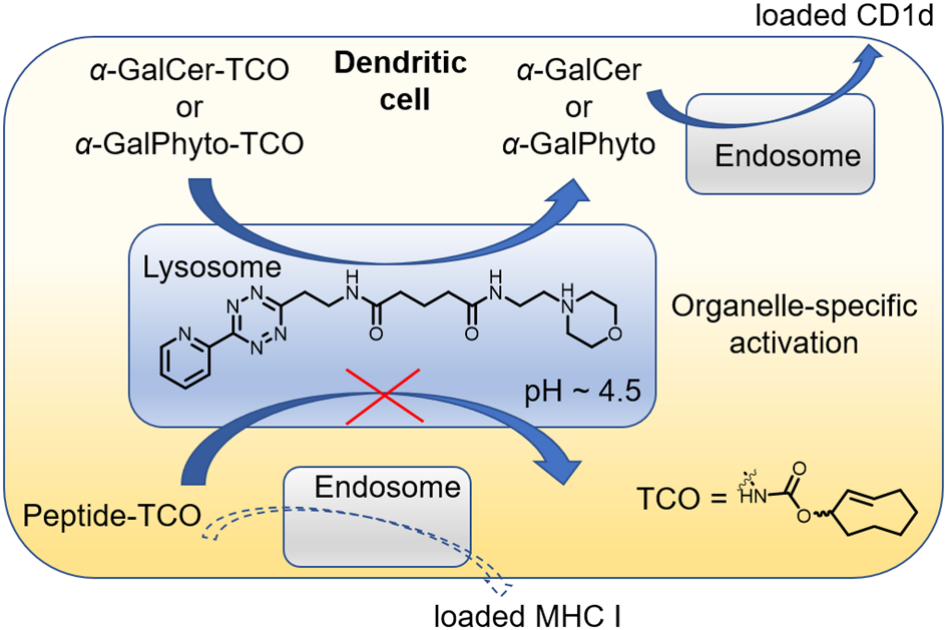
Lysosome-targeted Tz reagent enables organelle-specific activation of antigens in dendritic cells

The presentation of antigens by dendritic cells (DCs) plays a crucial role in immune responses [78]. Antigen presentation is a complex process, and the specific roles of individual organelles within this process remain the subject of ongoing debate. Investigating the contribution of lysosomes in antigen presentation, van Kasteren et al. developed a lysosome-targeted tetrazine probe to enable organelle-restricted activation of TCO-caged antigens [79]. Immune cell activation occurred only when the bioorthogonal deprotection reaction took place within the organelle. As employed in previous studies, a morpholine group was incorporated into the tetrazine probe to target the lysosome (Fig. 12).

Using this strategy, the study unveiled insights into the trafficking of glycolipid ligands such as α-galactosyl-ceramide (α-GalCer) and α-galactosyl phytosphingosine (α-GalPhyto), both of which serve as ligands for the CD1d receptor on DCs. Interestingly, these ligands were observed to pass through lysosomes before being presented by DCs. Conversely, the study demonstrated that CD8-activating long peptide antigens do not pass through lysosomes during cross-presentation on MHC-I molecules. This work is a notable example of how investigations employing modern bioorthogonal reactions at the level of individual organelles contribute to our understanding of immunological processes.

### Transition Metal Catalysis in Lysosomes

Ferritin (FTn) proteins can act as carriers for in situ nucleation of metal nanoparticles, forming what are known as nanozymes [80]. Sun and co-workers investigated the incorporation of various metals (Co, Fe, Mn, Rh, Ir, Pt, Au, Ru, and Pd) into the FTn cavity to form different nanozymes [81]. To validate the efficacy of the nanozymes, the researchers devised a fluorescence-based system employing caged probes. When successfully uncaged, the probes changed from nonfluorescent to fluorescent, a reaction catalyzed by the nanozymes. This approach enabled the cleavage reaction to be directly quantified through fluorescence detection. Among the screened candidates, Pd nanozymes were found to be the most promising, specifically cleaving the propargyl ether moiety. Remarkably, the Pd nanozymes displayed enzymatic activities not unlike horseradish peroxidase, particularly in acidic environments. Indeed, the ability of the nanozyme to cleave propargylic ether bonds was comparable to that of a mutated P450BM3 enzyme [82].

**Fig. 13:**
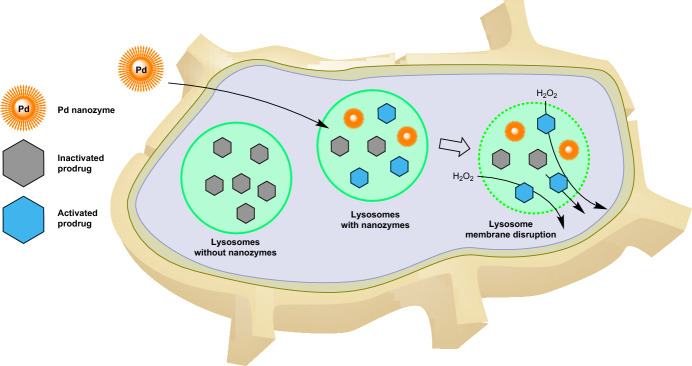
Lysosome-targeted nanozymes enabling organelle-specific prodrug activation accompanied by disruption of the lysosomal membrane and release of drug molecules into the cytosol

Pd nanozymes have shown promise in targeting cancer cells, such as MDA-MB-231 breast cancer cells, and have been found to localize primarily within cellular lysosomes. Given that Pd nanozymes display high enzymatic activity in the acidic environments typical of lysosomes [83], the researchers investigated their potential therapeutic utility. They found that Pd nanozymes can generate free radicals within lysosomes, leading to the disruption of lysosomal membranes. This disturbance could facilitate the release of lysosome-entrapped therapeutic agents into the cytoplasm, enabling them to exert their beneficial effects (Fig. 13).

Further investigations by the group centered on the proficiency of Pd nanozymes in deprotecting propargyl ether groups from various therapeutic compounds, including hydroxycamptothecin (HCPT). The team convincingly demonstrated that Pd nanozymes efficiently activated prodrugs within tumor cells. Addressing delivery challenges, they developed a liposome-based system that combined Pd nanozymes and pro-HCPT. This system demonstrated prolonged circulation in the bloodstream and precise targeting of tumor sites. Leveraging the pH-responsive properties of the liposomes, activation occurred under acidic conditions mimicking the lysosomal milieu. In vivo studies underscored the potential of these liposomes for therapeutic applications, particularly in targeting tumors.

## Reactions in Mitochondria

Mitochondria are pivotal organelles with a myriad of functions, ranging from energy production and cellular metabolism to signaling pathways that impact cell survival and apoptosis. Unraveling the dynamics of mitochondria through bioorthogonal reactions holds promise for advancing our understanding of human health, disease mechanisms, and potential therapeutic interventions. This section highlights recent advances in the use of bioorthogonal chemistry to decipher the pivotal contributions of mitochondria to cellular function and therapeutic purposes.

### Click Reactions in Mitochondria Based on Enrichment of Reagents

The mitochondrial membrane potential (Δψ_m_), which is central to the many functions of the organelle, plays a key role in driving ATP synthesis through oxidative phosphorylation [84]. The value of Δψ_m_ (≅ 120–180 mV, negative on the inside) is established by the balance between generation and consumption of electrons during ATP synthesis. In this context, Δψ_m_ serves as a sensitive indicator of cellular energetics. An increase in Δψ_m_ can be caused by diminished ATP synthesis or an upregulation in proton pumping, changes characteristic of apoptosis and cancer [85]. An elevation in Δψ_m_ is also associated with the production of mitochondrial reactive oxygen species (ROS), particularly in pathological scenarios and redox signaling [86]. Therefore, the ability to accurately quantify subtle changes in Δψ_m_ in vivo is important for the progression of novel diagnostic tools and therapies.

**Fig. 14:**
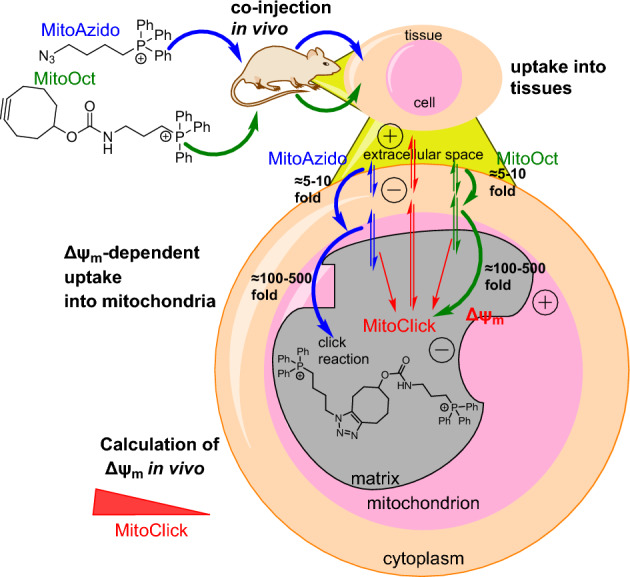
Molecular structures of MitoAzido and MitoOct, showcasing their cellular uptake triggered by Δψ_p_ and subsequently into mitochondria in response to Δψ_m_. The ensuing SPAAC ligation results in the formation of a regioisomeric mixture of MitoClick products detectable by LC–MS/MS

Murphy and colleagues employed two mitochondria-targeted probes, MitoAzido and MitoOct, each containing a triphenylphosphonium (TPP) lipophilic cation. This cation drives the cellular accumulation of the probes in response to Δψ_m_ and the plasma membrane potential (Δψ_p_) [87]. Once the probes accumulate within the mitochondria, which leads to a local increase in concentration, they undergo an SPAAC reaction to form a MitoClick product. In this context, the extracellular generation of the click product is mitigated because of the low concentration of the reagents. This renders the formation of the SPAAC product exceptionally sensitive, allowing for the detection of the subtlest alterations in both Δψ_m_ and Δψ_p_. These changes can be quantified using liquid chromatography-tandem mass spectrometry (LC-MS/MS) (Fig. 14).

The effectiveness of this method has been demonstrated by examining Δψ_m_ changes in mice. The swift mitochondrial accumulation of TPP probes within the heart led to the immediate appearance of MitoClick, potentially offering a means to study Δψ_p_ and Δψ_m_ changes in vivo.

Overcoming obstacles associated with mitochondria-targeted therapies, such as the necessity for specialized delivery strategies, and the limitations in confirming mitochondrial uptake due to sensitivity issues [88], is of paramount importance. To address these challenges, the same research group employed MitoOct [87], which selectively undergoes a SPAAC reaction with the delivered compound, enabling the detection of the resultant click product by mass spectrometry [89].

To validate this approach, a 29-residue mitochondrial targeting sequence (MTS) from the COX8 cytochrome *c* oxidase subunit (COX8-Z) [90] was used to target the mitochondria through the protein import machinery. Subsequently, COX8-Z was allowed to react with MitoOct, leading to the formation of COX8-Click, which was detected using matrix-assisted laser desorption ionization time-of-flight mass spectrometry (MALDI-TOF MS). As a proof of concept, the researchers also evaluated the mitochondrial uptake of a COX8 peptide conjugated with a peptide nucleic acid (PNA) tetramer (GCTA) linked to a C-terminal azide (COX8–PNA–Z). Notably, COX8–PNA–Z yielded COX8–PNA–Click upon treatment with MitoOct. The method was deemed successful in monitoring the progress of mitochondrial targeting, confirming uptake of the molecule of interest by the mitochondria.

**Fig. 15:**
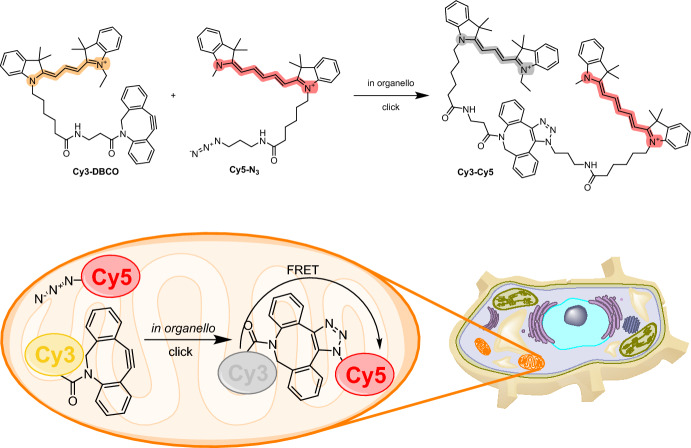
Structures of the mitochondriotropic Cy3–DBCO and Cy5–N3 probes. The two compounds react in cell mitochondria, resulting in the formation of a FRET click product

To study the delivery of molecules to mitochondria, Helm and colleagues exploited the intrinsic propensity of cyanine dyes to accumulate within this organelle. The authors established a Förster resonance energy transfer (FRET)-based system [91], focusing on a SPAAC reaction of two mitochondriotropic molecules. They synthesized a cyanine-based dye functionalized with dibenzocyclooctyne (Cy3–DBCO) as well as a Cy5-functionalized azide derivative (Cy5–N3). The chemical reaction between these components yielded a dicationic SPAAC product, Cy3–Cy5. This product exhibited unique properties that were absent from the individual constituents of the dye, namely modified membrane permeability and a distinct FRET effect (Fig. 15). The spectrum of the SPAAC product exhibited near-complete quenching of the donor emission and high acceptor emission when excited at the excitation wavelength of the Cy3 donor. These spectral characteristics were attributed to FRET, which was used as a readout to confirm successful coupling of the dyes in the mitochondria.

By imaging rat brain endothelial cells (RBE4) exposed to these dyes, the researchers verified the localization of the dyes within the mitochondria. The staining exhibited a high degree of specificity for mitochondria and was reversible upon disruption of the mitochondrial membrane potential. The FRET signal increased in the mitochondria during the first 4 h of incubation. Interestingly, the kinetics of the SPAAC reaction within the mitochondria were distinct from reactions conducted in the cuvette, possibly because of the enrichment of the probes in the organelle. Additionally, the authors performed the same experiment with reversed functionalities to confirm their interchangeability. Curiously, the formation of the specific product in this case resulted in a slower reaction compared to the previously described configuration.

### Enrichment-Triggered Prodrug Activation

**Fig. 16:**
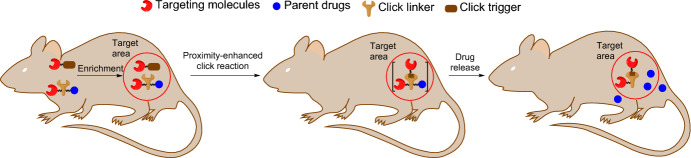
Schematic illustration of a strategy based on enrichment-triggered prodrug activation

Shifting the focus from organelle-selective imaging, Wang et al. devised a strategy for prodrug activation based on bioorthogonal reaction kinetics [92]. A targeting TPP moiety was employed to direct probes to the mitochondria, which led to a significant 100- to 500-fold enrichment of nontoxic prodrugs within the organelle. This triggered an efficient click reaction, which initiated the subsequent release of the biologically active molecule (Fig. 16).

**Scheme 4:**
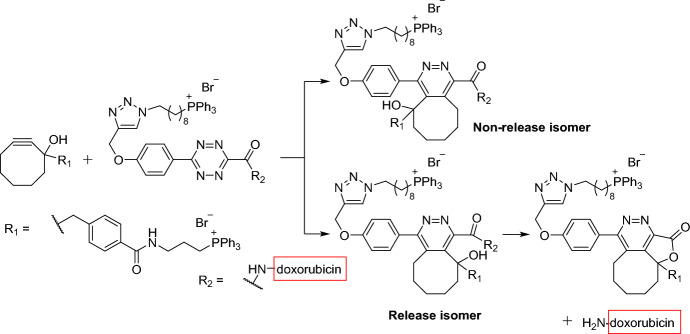
Tetrazine–cyclooctyne pair enables drug release in mitochondria after intramolecular lactonization

The authors designed two pairs of prodrug-releasing trigger partners. The first pair, consisting of tetrazine and cyclooctyne, was used for the click reaction and subsequent lactonization, resulting in the effective release of the doxorubicin. The second pair, based on the cyclopentadienone-strained alkyne system, was employed to achieve the controlled release of carbon monoxide (CO). For the tetrazine–cyclooctyne pair, > 80% of the drug was released within 48 h. This indicates that the regiochemistry favored the release isomer product, leaving the hydroxyl group on the same side as the doxorubicin-linked amide group (Scheme 4). The authors demonstrated that different R1 groups on the alkyne component were able to modulate the reaction rate by > 30-fold. Furthermore, altering the temperature from room temperature to 37 °C resulted in an additional three- to tenfold increase in reaction rates. Evaluating the enrichment strategy in HeLa cells, the authors confirmed that mitochondrial enrichment enhanced drug release. As expected, the reaction was sluggish in the absence of enrichment, resulting in the release of only a small amount of the active drug molecules.

**Scheme 5:**
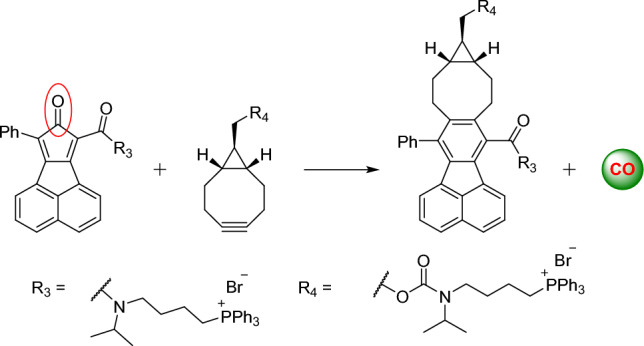
Enrichment and CO release system based on an intramitochondrial cyclopentadienone–BCN click reaction

The enrichment and release strategy was subsequently compared with a different chemical system, using the cyclopentadienone−BCN pair to facilitate CO delivery and release within mitochondria (Scheme 5). Starting compounds, both incorporating the TPP moiety, were first synthesized. The cyclopentadienone compound featured a naphthalene group attached to the dienone ring, allowing for visualization following the click reaction. The released CO induced a distinct blue fluorescence in RAW264.7 macrophages, which colocalized with the fluorescence of the mitochondria tracker. This confirmed the presence of the released CO in the mitochondria. Moreover, when cells were pretreated with these two compounds and then exposed to bacterial lipopolysaccharide, they exhibited suppressed expression of tumor necrosis factor (TNF). In contrast, control compounds lacking the TPP moiety had no influence on TNF production. Compared to other CO prodrugs [93, 94], this system had a lower IC_50_ value (~ 5 μM) and almost completely suppressed TNF production at 10 μM. The system even proved effective in a mouse model.

**Fig. 17:**
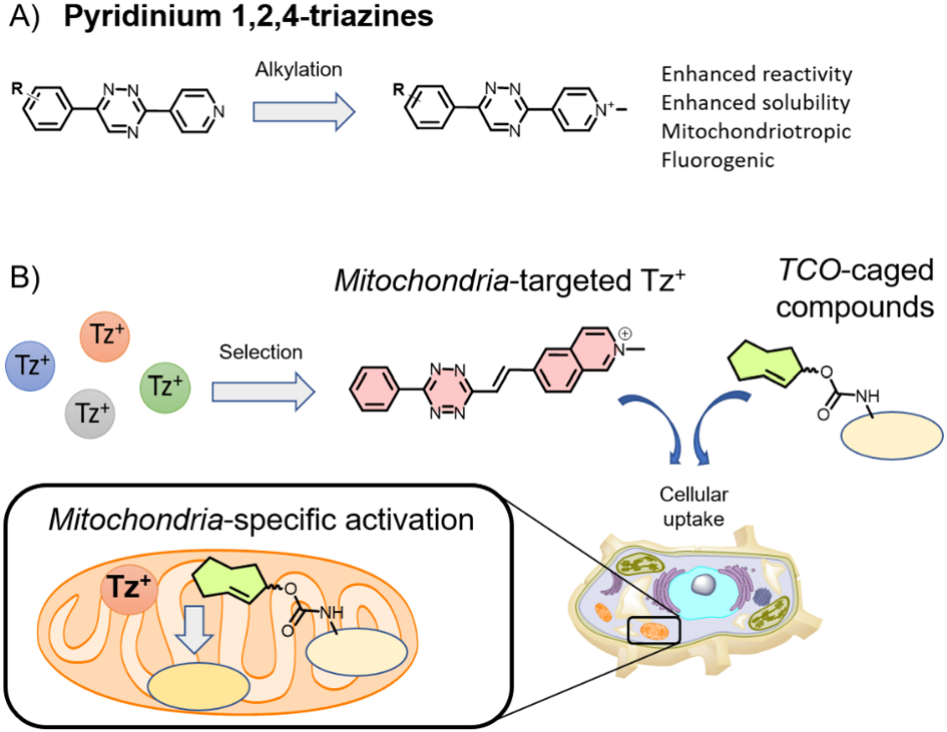
**a** Positively charged pyridinium 1,2,4-triazines exhibit spontaneous mitochondrial accumulation within live cells. **b** Targeting tetrazines (Tzs) to this organelle facilitates intramitochondrial prodrug activation based on an IEDDA chemical reaction and a release reaction sequence

The use of diverse targeting moieties is a common strategy for localizing compounds within specific cellular compartments. Siegl et al. devised an alternative approach based on their unexpected observation that 1,2,4-triazines bearing a pyridinium moiety have an intrinsic capacity to localize in the mitochondria of live cells (Fig. 17A) [95].

These probes combine fluorogenic properties with an innate ability to react with TCOs, allowing for efficient and selective fluorescent labeling of the organelle. Based on these observations, Dzijak et al. investigated a series of 1,2,4,5-tetrazines incorporating different positively charged groups. Their aim was to identify bioorthogonal Tz probes intrinsically capable of localizing in live cell mitochondria [96]. One of the compounds, containing an isoquinolinium moiety, demonstrated excellent localization in the organelle. This mitochondriotropic Tz probe was then utilized in an organelle-specific click-to-release reaction. The compound was able to activate a TPP-tagged niclosamide prodrug specifically within the organelle, leading to the depolarization of mitochondrial membrane potential and subsequent cell death (Fig. 17B). As a result, the targeted delivery and activation of the drug molecule within the mitochondria substantially enhanced its biological efficacy, surpassing the unmodified parent drug by > 100-fold. This study demonstrates the potential for optimizing commonly employed bioorthogonal reagents. As well as restricting their intracellular distribution, their physicochemical properties can be finely tuned for selective organelle targeting. By exploiting their bioorthogonal reactivity, these probes can be deployed to precisely manipulate biological processes at the subcellular level.

### Detection of Mitophagy by Enrichment-Triggered Click Reaction

**Fig. 18:**
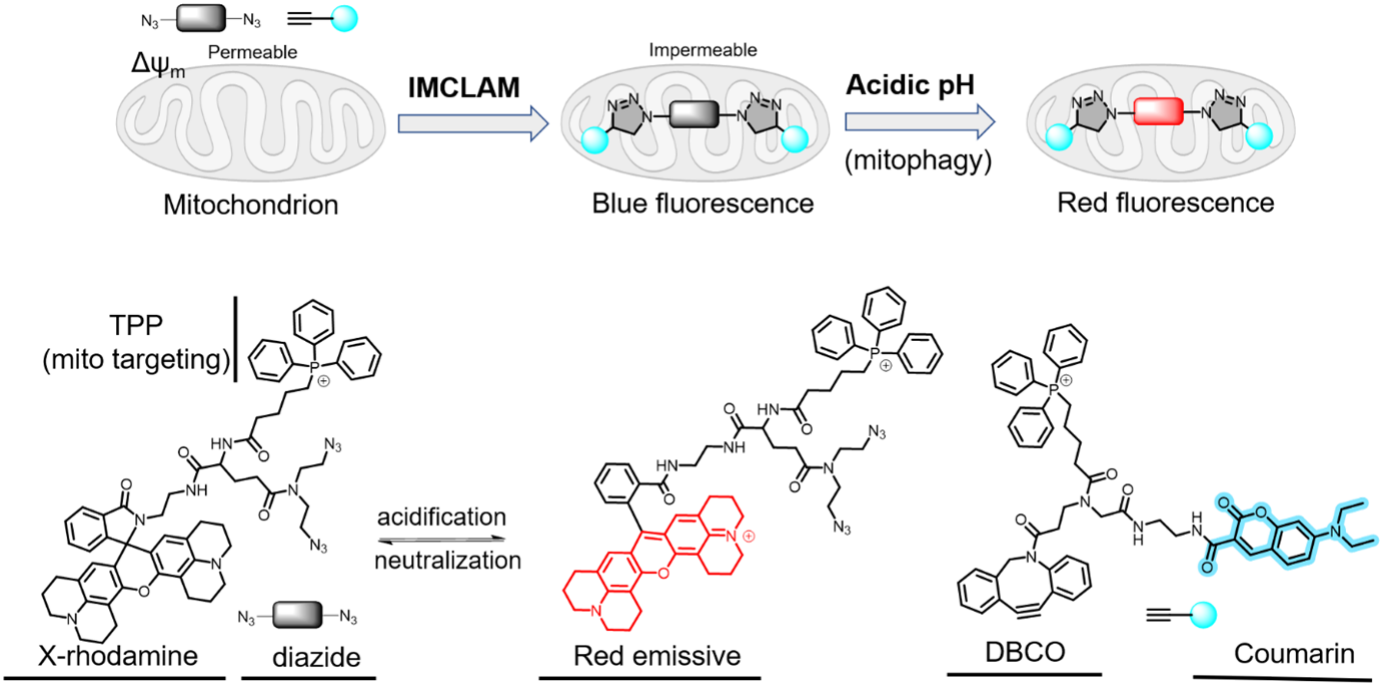
Intramitochondrial click reaction for mitophagy analysis

Mitophagy is a process that involves the degradation and turnover of dysfunctional mitochondria within lysosomes [97]. Targeting this process is of potential value for pharmacological intervention [98]. To this end, Shi et al. devised a novel technique called intramitochondrial CLICK for assessing mitophagy (IMCLAM). This method allows dysfunctional mitochondria to be detected independently of changes in mitochondrial membrane potential (Fig. 18) [99].

The technique leverages two probes, which accumulate in mitochondria based on mitochondrial potential. This accumulation results in the creation of a covalent click product notable for its large molecular size, which ensures it is retained within the organelle even after membrane depolarization. The formation of the click product involves a SPAAC reaction between a strained DBCO and an azide probe, both of which contain a mitochondria-targeting TPP moiety. Fluorescence imaging is an important element in mitophagy detection. In this case, the researchers reacted a rhodamine diazide probe with a DBCO–coumarin compound. In the presence of a pH-activatable lactam rhodamine dye, the click sensor selectively generates a red fluorescent signal once mitochondria enter the lysosomes, indicating mitophagy. Using this method, the authors were able to optically trace stressed mitochondria and perform fluorescence turn-on imaging to observe mitophagy, demonstrating its value in identifying mitophagy-inducing pharmacological compounds.

### Detection of Autophagic and Endocytic Fluxes

**Fig. 19:**
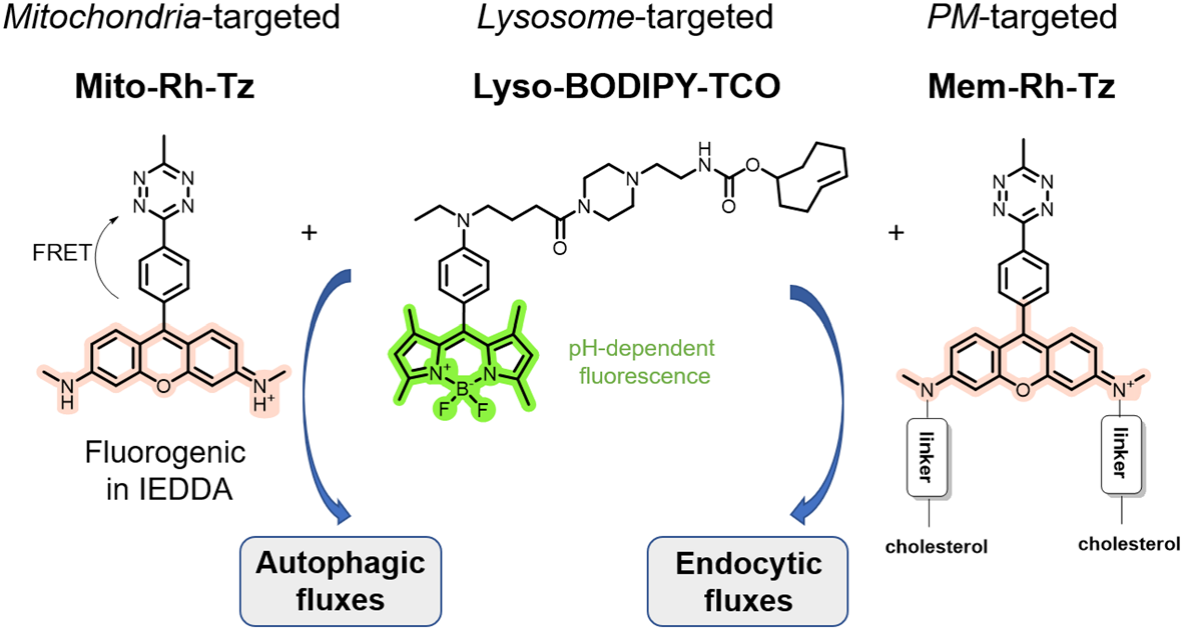
Bioorthogonal probes enable autophagic and endocytic fluxes to be monitored via an intraorganellar IEDDA reaction

The expression of various cell surface markers is tightly regulated by cellular events such as autophagic and endocytic fluxes. Monitoring these fluxes can provide useful information for disease diagnosis. In addition, understanding the correlation between autophagic and endocytic fluxes and the expression of cell surface proteins is particularly important for cancer treatment [100]. Specifically, markers like major histocompatibility complex class I (MHC-I) and programmed cell death ligand 1 (PD*–*L1) play pivotal roles in cancer immunotherapy [101, 102]. Jiang and colleagues developed an innovative system based on a bioorthogonal IEDDA reaction to monitor and correlate fluxes with the expression of therapeutic targets [103]. For flux monitoring, they constructed a lysosome-targeted probe (Lyso*–*BODIPY*–*TCO), featuring a BODIPY fluorophore, a TCO dienophile, and piperazine as the lysosome-targeting moiety. Notably, this probe is nonfluorescent at neutral pH but becomes fluorescent in the acidic environment of lysosomes. In addition, two tetrazine probes were employed: one harboring a mitochondria-targeted rhodamine dye (Mito*–*Rh*–*Tz) and the other containing cholesterol groups for plasma membrane targeting (Mem*–*Rh). Both tetrazine probes were designed to emit red fluorescence upon reacting with TCO. This method is based on the formation of a fluorescent click product. In this case, Lyso*–*BODIPY*–*TCO can either react with Mito*–*Rh during autophagy-induced lysosome–mitochondria fusion (mitophagy) or with Mem*–*Rh*–*Tz upon endocytosis-driven endosome–lysosome fusion (Fig. 19).

By using these organelle-specific probes, fluctuations in fluorescent signals allow autophagic and endocytic fluxes to be visualized directly. Finally, the technique was used to examine the role of fluxes in modulating the expression of MHC-I and PD-L1. These studies show that inhibitors of autophagy and endocytosis have great potential to enhance the efficiency of current immunotherapies.

### Reactive Mitochondriotropic Molecules

**Fig. 20:**
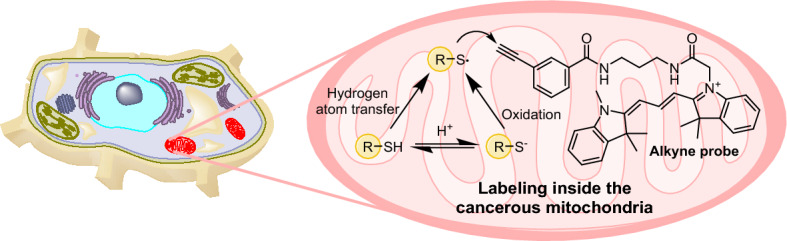
Example of a thiol–yne coupling reaction in mitochondria

Heinrich and colleagues studied radical thiol–yne coupling reactions in live cells [104]. The researchers employed a diverse array of fluorescent alkynes and alkenes, derived from rhodamine, cyanine, fluorescein, and doxorubicin, to explore their reactivity with thiols under oxidative conditions simulating those found in cancerous mitochondria [105]. In vitro investigations were carried out in cancer cells, which were incubated with the probes and control compounds. The cells were then analyzed by flow cytometry. The phenyl alkynes showed the most pronounced reactivity with endogenous thiols, highlighting their value as probes in intramitochondrial thiol–yne coupling (Fig. 20). Colocalization experiments using confocal microscopy confirmed the accumulation of rhodamine, fluorescein, and cyanine probes in mitochondria. However, doxorubicin displayed a significantly different distribution pattern, consistent with its well-known inability to permeate mitochondria. Additionally, alkyne-containing probes showed enhanced toxicity compared to the alkyne-free control compounds, indicating the effect of the radical thiol–yne reaction on the cell viability of cancer cells. Moreover, flow cytometry analysis revealed a preference for covalent binding to cancerous cells over noncancerous cells. This was attributed to a higher concentration of mitochondrial ROS in cancer cells [106].

The authors concluded that the combination of compound-directed accumulation in mitochondria and stimuli-responsive reactivity is a promising strategy for inducing selective mitochondrial dysfunction in cancer cells. This opens up new opportunities for the development of mitochondria-targeted therapeutics and bioactive molecules in cancer treatment and other biomedical applications.

**Fig. 21:**
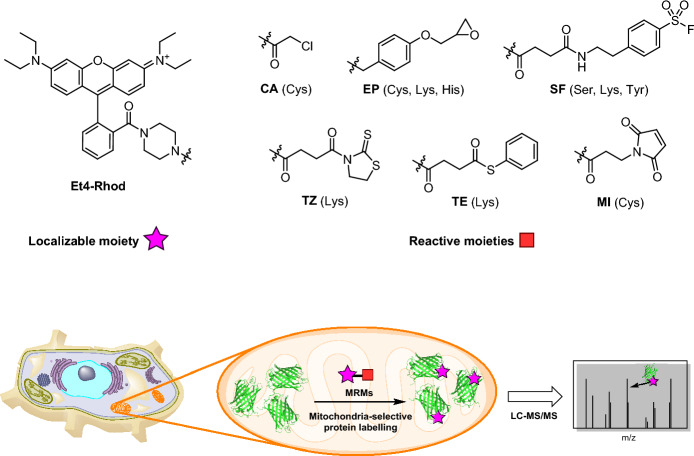
Examples of mitochondria-localizable reactive molecules and an overview of the strategy used for mitochondria-selective proteomics

In the field of proteomics, Hamachi and colleagues developed organelle-reactive probes known as mitochondria-localizable reactive molecules (MRMs) [107]. The design of these MRMs involves the incorporation of two main moieties: a mitochondria-localizing moiety and a chemically reactive moiety. For the localization part, the researchers used tetraethyl-rhodamine (Et4-Rhod). This compound not only enables the visualization of MRM localization through fluorescence imaging but also serves as a marker for mitochondrial localization. As for the reactive moiety, the researchers explored eight different reactive functionalities, each exhibiting varying selectivity for amino acids. These included chloroacetyl (CA) and maleimide (MI) for targeting thiol groups of cysteines [108, 109], thiophenyl ester (TE) and thiazolidinethione (TZ) for reacting with amino groups of lysine [110], epoxide (EP) for reacting with nucleophilic amino acids such as Cys, Lys, and His [111], and sulfonyl fluoride (SF) for labeling active sites of Ser proteases as well as Lys and Tyr residues (Fig. 21) [112, 113]. Although the authors did not employ authentic bioorthogonal reactions, their strategy nonetheless facilitated targeted analysis of the mitochondrial proteome. Indeed, they successfully applied the method to analyze various biological samples, including HeLa cells, primary neurons, and brain slices.

### Transition Metal Catalysis in Mitochondria

The first example of an abiotic, bioorthogonal catalytic system tailored for subcellular compartments in living cells was developed by Tomás-Gamasa et al. They explored a range of ruthenium complexes designed to accumulate within the mitochondria of mammalian cells while retaining their ability to react with exogenous substrates in a bioorthogonal manner [114].

**Fig. 22:**
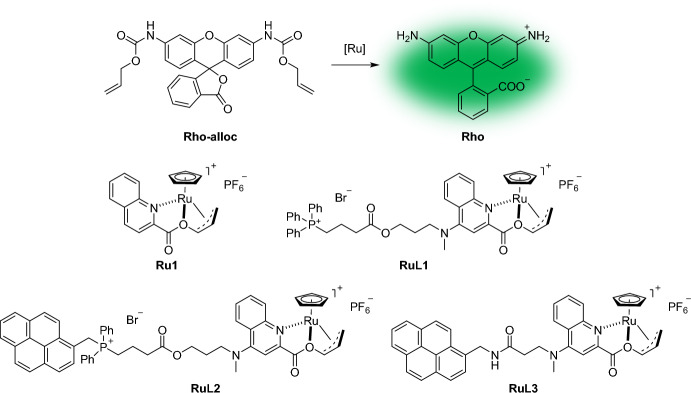
Schematic presentation of the uncaging reaction and catalyst structures examined in the study

The first RuL1 complex contained a modified 2-quinolinecarboxylate ligand tethered to a relatively hydrophobic alkyl chain, which was linked to a TPP group for mitochondrial accumulation. A control catalyst, Ru1 without the TPP group, was also synthesized. The catalytic efficacy of the Ru complexes in removing the allyloxycarbonyl (alloc) group was assessed using protected, fluorogenic rhodamine (Rho-alloc), which upon deprotection yields fluorescent rhodamine (Rho) (Fig. 22).

To visualize the intracellular presence of the metal complexes, a fluorescent pyrene moiety was incorporated into the TPP moiety (RuL2) instead of one of the phenyl rings. A control compound lacking the TPP moiety (RuL3) was also prepared. Upon incubation with HeLa cells, RuL2 exhibited a distinct blue intracellular fluorescence within the mitochondria, which colocalized with the mitochondrial dye tetramethylrhodamine ethyl ester (TMRE). In contrast, RuL3 displayed a different staining pattern, characterized by a concentration of fluorescence predominantly in the perinuclear region.

Inductively coupled plasma mass spectrometry (ICP-MS) analysis of ruthenium content in isolated mitochondria indicated that the RuL2 complex had the highest accumulation. Consequently, the presence of the pyrene group enhanced its cellular and mitochondrial retention when compared to RuL1. This highlights the effectiveness of the phosphonium-pyrene unit, which serves as both a mitochondrial targeting element and a fluorescent marker.

Catalytic activity within cells was demonstrated through the deprotection of Rho-alloc, leading to the emission of green fluorescence from the liberated Rho. This fluorescence matched the red fluorescence from the TMRE dye as well as the blue fluorescence emitted by RuL2. For RuL3, the overlay between Rho fluorescence and the TMRE signal was notably weaker. Similar results were observed in adenocarcinomic human alveolar basal epithelial cells (A549 cells).

**Fig. 23:**
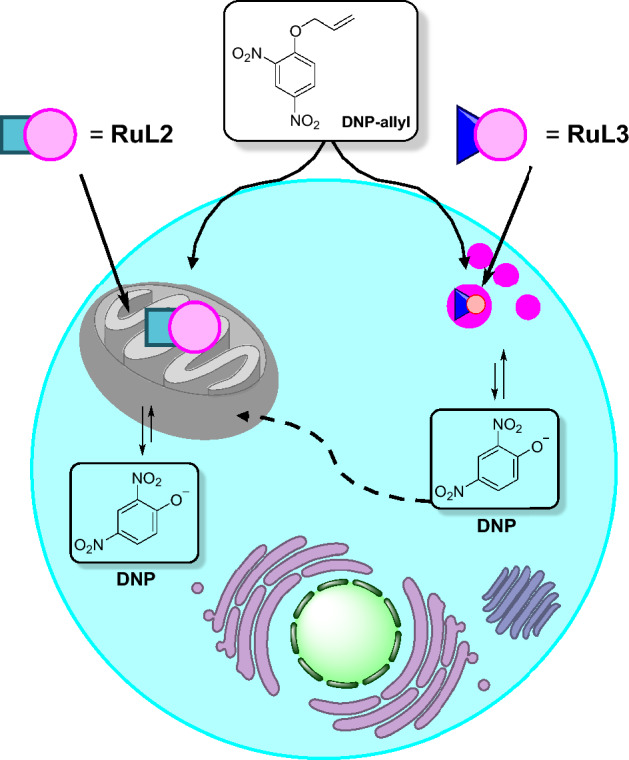
Schematic representation of DNP-allyl uncaging with RuL2 and RuL3

Lastly, the catalytic system was employed to activate a mitochondrial uncoupler, 2,4-dinitrophenol (DNP), from its allyl-caged form, called DNP-allyl. The Ru-promoted reaction led to depolarization of membrane potential, which in turn influenced ATP production (Fig. 23) [87, 115]. The study demonstrates that the same degree of mitochondrial membrane depolarization achievable with a toxic concentration (> 500 µM) of active DNP can be attained with a lower, nontoxic concentration (150 µM) of the DNP-allyl precursor in conjunction with RuL2.

**Fig. 24:**
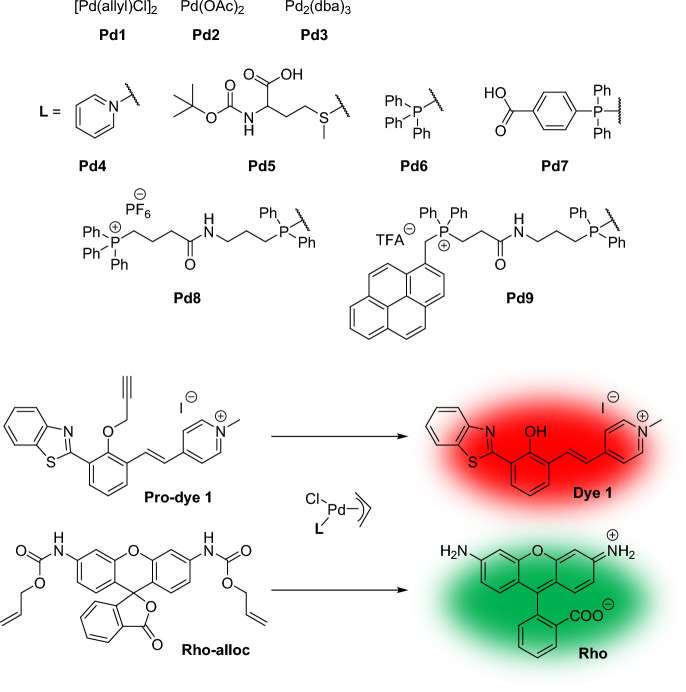
Propargyl and alloc group deprotection with Pd complexes

Building on previous examples demonstrating intracellular activity of Pd nanoparticles [116], Martínez-Calvo et al. investigated a series of Pd complexes (Pd1–Pd9) capable of triggering depropargylation and deallylation reactions in both cell lysate and live cells (Fig. 24) [117]. Employing fluorogenic propargyl- or allyl-caged dyes, the new catalysts were evaluated within living cells. Of these complexes, Pd6, Pd7, and Pd8, which incorporate phosphine ligands, exhibited robust red fluorescence in Vero cells, indicating a balanced interplay between reactivity and stability within the cellular environment. Additionally, these reactions were effectively conducted in HeLa cells. Intriguingly, pretreatment of cells with Pd reagents followed by the addition of the pro-fluorophore failed to generate any fluorescent signal, presumably because of the conversion of Pd complexes to inactive species. In another study, it was observed that even in simple systems such as phosphate-buffered saline, transition metal catalysts underwent partial deactivation [118].

Rho-alloc was also successfully decaged within living HeLa cells. While Pd1–Pd5 yielded only minimal intracellular fluorescence during deprotection reactions, Pd6–Pd8 elicited strong green fluorescence. Similar to the results observed with ruthenium complexes [114], Pd9 containing the pyrene group efficiently colocalized with MitoTracker in mitochondria, enabling an organelle-specific reaction.

**Fig. 25:**
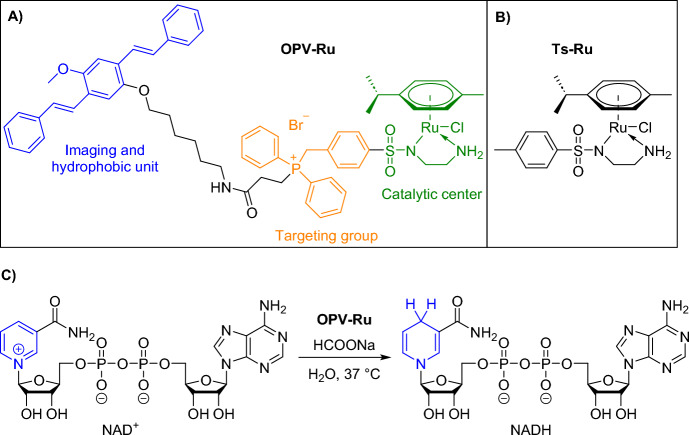
Structures of **a** OPV-Ru and **b** Ts-Ru. **c** Transfer hydrogenation reaction of NAD^+^ with HCOONa as a hydrogen source for NADH production

Another example of transition metal catalysis in the mitochondria of live cells was demonstrated by Dai et al. They introduced a multifunctional, ruthenium-coordinated oligo(*p-*phenylenevinylene) (OPV*–*Ru) metal catalyst designed for intracellular transfer hydrogenation reactions [119]. The hydrogenation of nicotinamide adenine dinucleotide (NAD^+^) to its reduced form (NADH) was selected as a model reaction inside the cells. NAD^+^/NADH plays an important role in regulating many metabolic pathways, including glycolysis, the citric acid cycle, and oxidative phosphorylation [120, 121]. Additionally, it accurately reflects the redox state of a mammalian cell [122]. The catalytic system consists of the following components: a Noyori-type ruthenium catalyst for the transfer hydrogenation; benzyldiphenylphosphonium for mitochondria targeting (similar to TPP); three benzene rings, which serve as a fluorescent moiety for cell imaging; and a six-carbon atom chain for hydrophobicity, which provides the driving force for self-assembly in water (Fig. 25).

Using UV spectroscopy, the researchers assessed catalytic activity in the transfer hydrogenation of NAD^+^ to NADH in aqueous conditions at 37 °C; HCOONa served as the hydrogen source. Increased quantities of OPV-Ru or higher HCOONa concentrations corresponded to higher yields of NADH and enhanced reaction rates. A comparison between aggregation and catalytic efficiency was drawn with (*p*-cymene)Ru(TsEn)Cl (Ts-Ru), which lacks self-assembly capability. This comparison unveiled a significant time discrepancy: OPV*–*Ru reached the transfer hydrogenation endpoint in 45 min, whereas Ts*–*Ru required over 600 min. Isothermal titration calorimetry measurements indicated that NAD^+^ exhibited a preference for binding to OPV*–*Ru nanoparticles rather than to the dispersed Ts-Ru catalyst. Moreover, OPV*–*Ru nanoparticles carrying positive charges proved adept at bringing the negatively charged NAD^+^ and catalytic centers into closer proximity, providing a local microenvironment with significantly enhanced catalytic efficiency.

OPV-Ru was able to enter A2780 human ovarian cells within 1 h, where the compound colocalized with MitoTracker Red FM dye and LysoTracker Red DND-99 dye, confirming its presence in both organelles. After 12 h of incubation, OPV-Ru remained present in both organelles. However, after 24 h, its presence in mitochondria diminished despite remaining in lysosomes. Cells treated with OPV*–*Ru and HCOONa exhibited a 23% decrease in NAD^+^ content compared to controls after 1 h. After 12 h, a relatively stable NAD^+^ to NADH ratio was achieved, pointing to the establishment of an equilibrium between NAD^+^ and NADH.

**Fig. 26:**
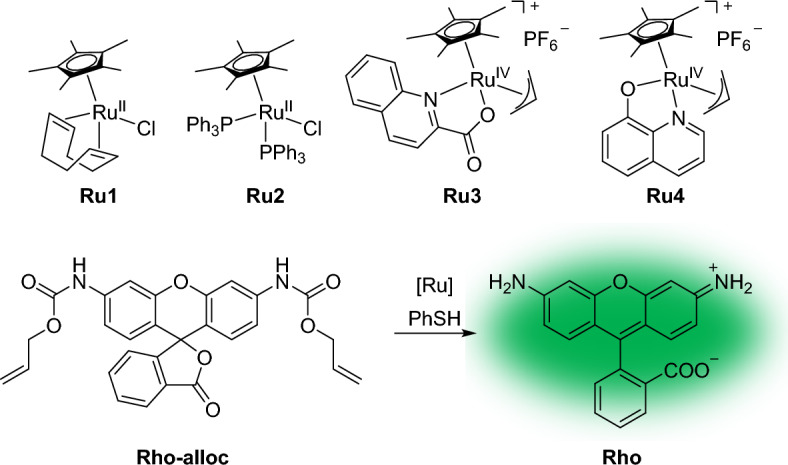
Ru(II) and Ru(IV) complexes screened for their allylcarbamate cleavage of Rho-alloc to fluorescent Rho

The performance of transition metal catalysts in living cells depends on many factors. The observed variations in the intracellular efficiency of organometallic reactions prompted Nguyen et al. [123] to conduct an in-depth analysis of metal-promoted reactions in living cells using single-molecule fluorescence microscopy (SMFM). The authors employed allylcarbamate cleavage reactions to monitor the reaction in real time. Their investigation centered around the determination of reaction locations, turnover frequency (TOF), and reactivity influenced by the cellular environment. Of the several ruthenium complexes they examined, Ru1–Ru4 displayed activity in the deprotection reaction of Rho*–*alloc, leading to the generation of fluorescent Rho in the presence of thiophenol (PhSH) (Fig. 26). The researchers later discovered that PhSH was not required for reactions occurring within cells, possibly because of the presence of endogenous thiols. The activity trend of the catalysts followed a general decrease in the order PBS > cell lysate > DMEM > DMEM/FBS because of an increase in potential nucleophiles in the media, which may have deactivated the Ru complexes. The DMEM/FBS medium, where the activity trend of the Ru catalysts was Ru4 > Ru3 > Ru2 ≈ Ru1, proved to be of particular relevance for living systems. This trend indicated that Ru1 and Ru2, the Ru(II) complexes, were more susceptible to inhibition than Ru4, which is a Ru(IV) species.

Using SMFM, the researchers successfully distinguished whether the reactions occurred inside or outside the cell. The frequency of spots corresponding to allylcarbamate cleavage reactions was evaluated using SMFM for all catalysts. Interestingly, they observed significant variability even among individual cells within the same sample.

To estimate the TOF of the reactions occurring within cells, the area spot frequency was divided by the concentration of the Ru complexes. Since the Ru complexes do not emit light, their concentrations were determined through ICP-MS measurements. The TOF order observed in non-PBS mixtures was maintained within the cell environment (Ru4 > Ru3 > Ru2 > Ru1).

Given that certain Ru species exhibit a strong affinity for mitochondria [124], investigations involving the mitochondria-specific dye MitoTracker Deep Red have been conducted using Ru3 or Ru4 in the presence of Rho*–*alloc. The results from these experiments indicate a higher occurrence of single-molecule events, such as allylcarbamate cleavage reactions, within mitochondria compared to the cytosol or other organelles. ICP-MS measurements revealed that approximately 3% of Ru was present in mitochondria, while approximately 97% remained in the cytosol. This distribution suggests that a substantial portion of Ru3 and Ru4 within the cytosol might exist in an inactive state, possibly because of coordinative inhibition by biomolecules or other species.

Based on the above examples, the presence of biological nucleophiles can pose challenges for the catalytic activity of transition metal catalysts within cells. Wang et al. introduced an innovative approach for organelle-specific CuAAC, enabling localized drug synthesis [125]. They devised a heterogeneous copper catalyst based on metal–organic frameworks (MOF*–*Cu), which displayed exceptional catalytic activity and stability. For mitochondrial targeting, they modified MOF*–*Cu with TPP to create MOF*–*Cu*–*TPP. Both MOF*–*Cu and MOF*–*Cu*–*TPP were efficiently endocytosed into human breast adenocarcinoma cells (MCF-7 cells) with minimal cytotoxic effects. To confirm mitochondrial localization, they incorporated fluorescein isothiocyanate (f-MOF*–*Cu*–*TPP) into MOF*–*Cu*–*TPP. The colocalization of f-MOF*–*Cu*–*TPP with the fluorescent signal from the mitochondrial red dye (MitoTracker Red CMXRos) confirmed the effective mitochondrial targeting by MOF*–*Cu*–*TPP.

**Scheme 6:**
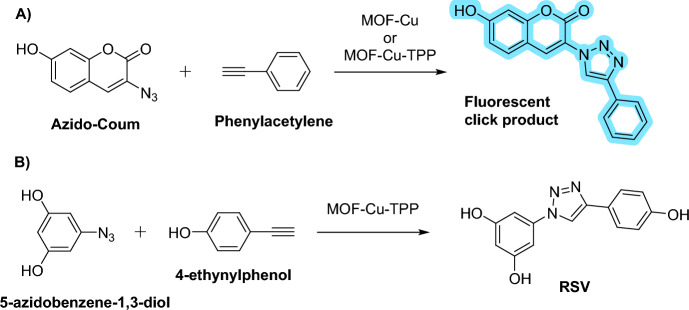
Intracellular CuAAC reaction catalyzed by MOF–Cu complexes leading to **a** fluorescent click products and **b** intramitochondrial synthesis of the resveratrol derivative

This catalytic system was optimized using a fluorogenic coumarin derivative. Subsequently, a derivative of resveratrol (RSV) was synthesized within the mitochondria from the respective azide and alkyne precursors. Notably, both precursors exhibited negligible toxicity up to 25 µM in MCF-7 cells. In contrast, the in situ synthesized RSV cytotoxic drug exhibited enhanced antitumor efficacy while circumventing the side effects associated with the parent resveratrol (Scheme 6).

The efficacy of this catalytic process was also demonstrated in *Caenorhabditis elegans*. No signs of morphological alternation or toxicity were observed. Additionally, the catalyst showed good biocompatibility in a mouse model, further underscoring its potential use in in vivo applications. This work illustrates that embedding active catalytic centers within a layer of the protective environment is an efficient way of building catalytic systems that can survive in the otherwise harsh environment of living organisms.

## Reactions in the Endoplasmic Reticulum

The endoplasmic reticulum (ER) plays vital roles in protein synthesis, folding, lipid metabolism, and cellular signaling. Understanding its functions is key to unraveling these cellular processes. Much like the targeting of other organelles, bioorthogonal reactions are essential tools for studying the ER and its functions. However, unlike lysosomes or mitochondria, there are far fewer examples of exclusive ER targeting. Given that the ER is distributed from the nucleus to the plasma membrane, targeting this signaling organelle can be challenging, often resulting in colocalization with other organelles like the mitochondria or Golgi. Recent examples of bioorthogonal reactions targeting the ER are given below.

### ER-Targeted Reagents in Proteomics

**Fig. 27:**
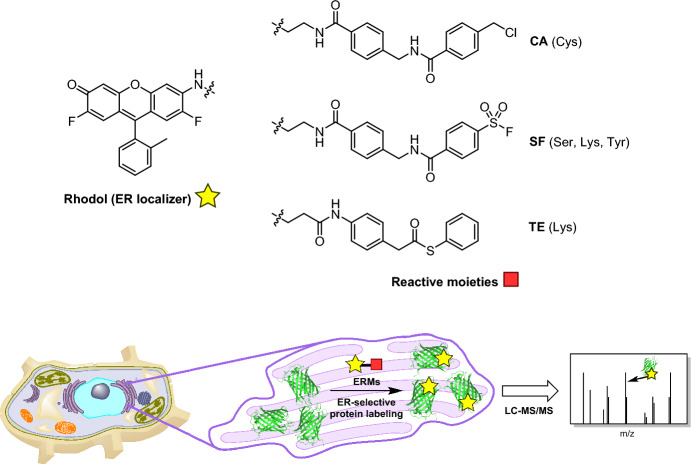
Set of ER-localizable reactive molecules for use in ER-targeted proteomics

Inspired by the approach they used to study mitochondrial proteomics [107], Hamachi and colleagues designed a series of probes to profile the ER proteome [126]. In this case, fluorinated rhodol was used for ER targeting in conjunction with various electrophilic warheads for covalent reaction with proteins (Fig. 27). The electrophiles studied were chloroalkane (CA), sulfonyl fluoride (SF), and thiophenyl ester (TE), each of which has unique amino acid preferences, ensuring broad coverage of proteins in the ER proteome. Additionally, the authors used the ER-targeted probes in parallel with other organelle-reactive molecules that target the nucleus and mitochondria [107, 127]. Their results demonstrated the potential for simultaneous tagging and identification of proteins across multiple organelles in a single sample.

### Dithiol-Activated Bioorthogonal Release Reaction

Elevated NO_2_^−^ levels are associated with antitumor effects and can induce protein nitration, DNA damage, and energy depletion through excessive ⋅NO_2_ production during oxidative and nitrosative stress. Traditional approaches that rely on inorganic nitrite salts for intracellular delivery have proven ineffective because of their rapid metabolism and potential side effects resulting from high-dose administration.

**Fig. 28:**
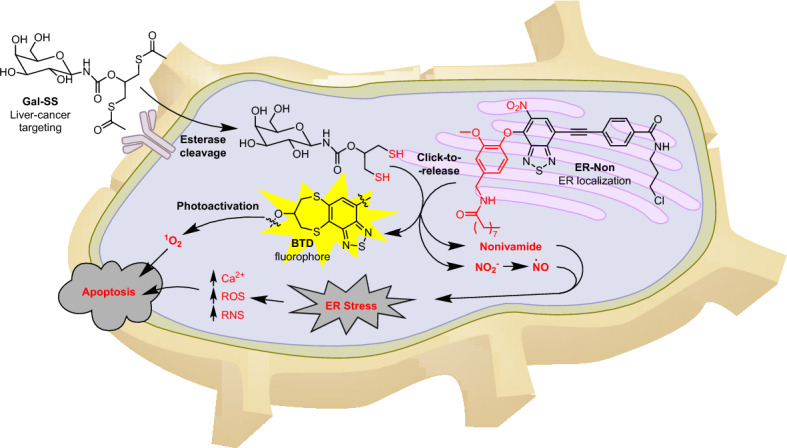
Schematic representation showing the working principles of a dithiol-activated bioorthogonal reaction

The therapeutic potential of NO_2_^−^ in various pathological conditions inspired Wang and colleagues to devise caged organic nitrite donors. They deployed the multifunctional ER-Non molecule, featuring the 2,1,3-benzothiadiazole (BTD) heterocycle, the anticancer drug nonivamide (Non), and an ER-targeting moiety, to specifically target the ER for NO_2_^−^ release [128]. The ER was localized by incorporating a 3-chloropropylamine moiety into the structure of the molecule [129]. Notably, these NO_2_^−^ donors exhibited exceptional stability against various biological species, while displaying a selective response to dithiols (Fig. 28).

Encouraged by this finding, the researchers developed the novel compound Gal-SS, which merges 1,3-dithioglycerol with β-galactosamine, to enhance the targeting of liver cancer. Decaging from the nitrite donors began with the enzymatic cleavage of the thioester moieties of Gal-SS upon intracellular entry, followed by a sequential tandem S_N_Ar/cyclization reaction with ER-Non. This cascade released NO_2_^−^ and the attached anticancer drug nonivamide.

This treatment resulted in the generation of ⋅NO species (generated by endogenous enzymes from NO_2_^−^), increased levels of both ROS and reactive nitrogen species (RNS), and depletion and transfer of Ca^2+^ ions from the ER to the cytosol. These effects contributed to the antitumor properties of nonivamide. In addition, elimination of the nitrite activated the BTD fluorophore, enabling cellular imaging. Enhanced antitumor effects were observed upon light exposure because of the photoreactivity of the compound. This combination of chemical and light treatment led to a substantial reduction in cell viability, highlighting the potential of integrating controlled chemotherapy with BTD-enhanced antitumor effects.

### Visualizing Changes in Membrane Potentials

**Scheme 7:**
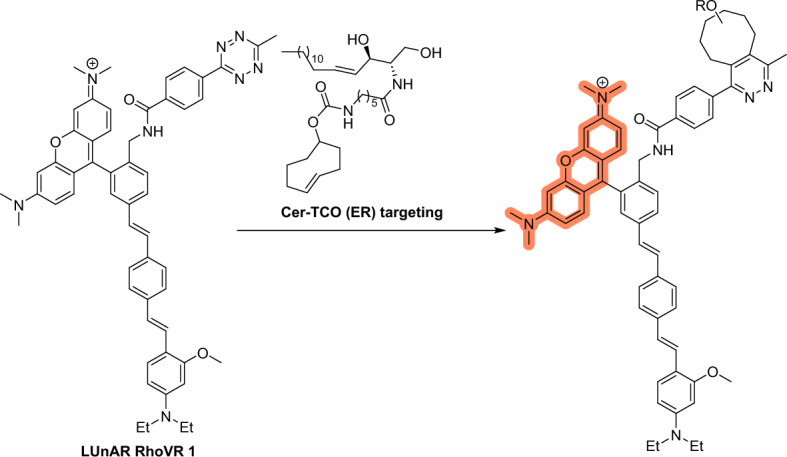
Structures of LUnAR RhoVR and Cer-TCO for targeted delivery of RhoVRs to organelles

Biological membranes maintain electrical potentials, which play a role in initiating numerous physiological processes. Methods such as optical and electrode-based approaches have been developed to assess plasma membrane potential [130]. However, targeting specific internal organelle membranes using voltage-sensitive dyes for the purpose of visualization is a challenging task. To that end, Miller et al. developed a system called ligation unquenched for activation and redistribution rhodamine-based voltage reporter (LUnAR RhoVR) [131], which they used to monitor changes in membrane potential within the ER of living cells. The authors demonstrated that the product of LUnAR RhoVR and *trans*-cyclooctene (TCO)-conjugated ceramide (Cer*–*TCO) is not only voltage-sensitive but can also target the ER of living cells (Scheme 7). This work represents an important breakthrough in that it demonstrates the response of the ER to changes in plasma membrane potential. With its ability to visualize the functional connection between plasma and ER membranes in patch-clamped cells, the LUnAR RhoVR system provides novel insights into cellular dynamics.

## Reactions on Cellular Membranes

Cellular membranes are important to diverse biological processes and essential for compartmentalization, signaling, and transport regulation. They serve as dynamic interfaces that define the boundaries of organelles and facilitate communication between intracellular compartments. By targeting cellular membranes, researchers can indirectly probe the localization, organization, and function of individual organelles. This strategic approach has been used in several studies. This section offers a comprehensive compilation of these studies, highlighting the role of bioorthogonal reactions in advancing our understanding of cellular membranes and their functional implications.

### Targeting Membranes in Advanced Microscopy

**Fig. 29:**
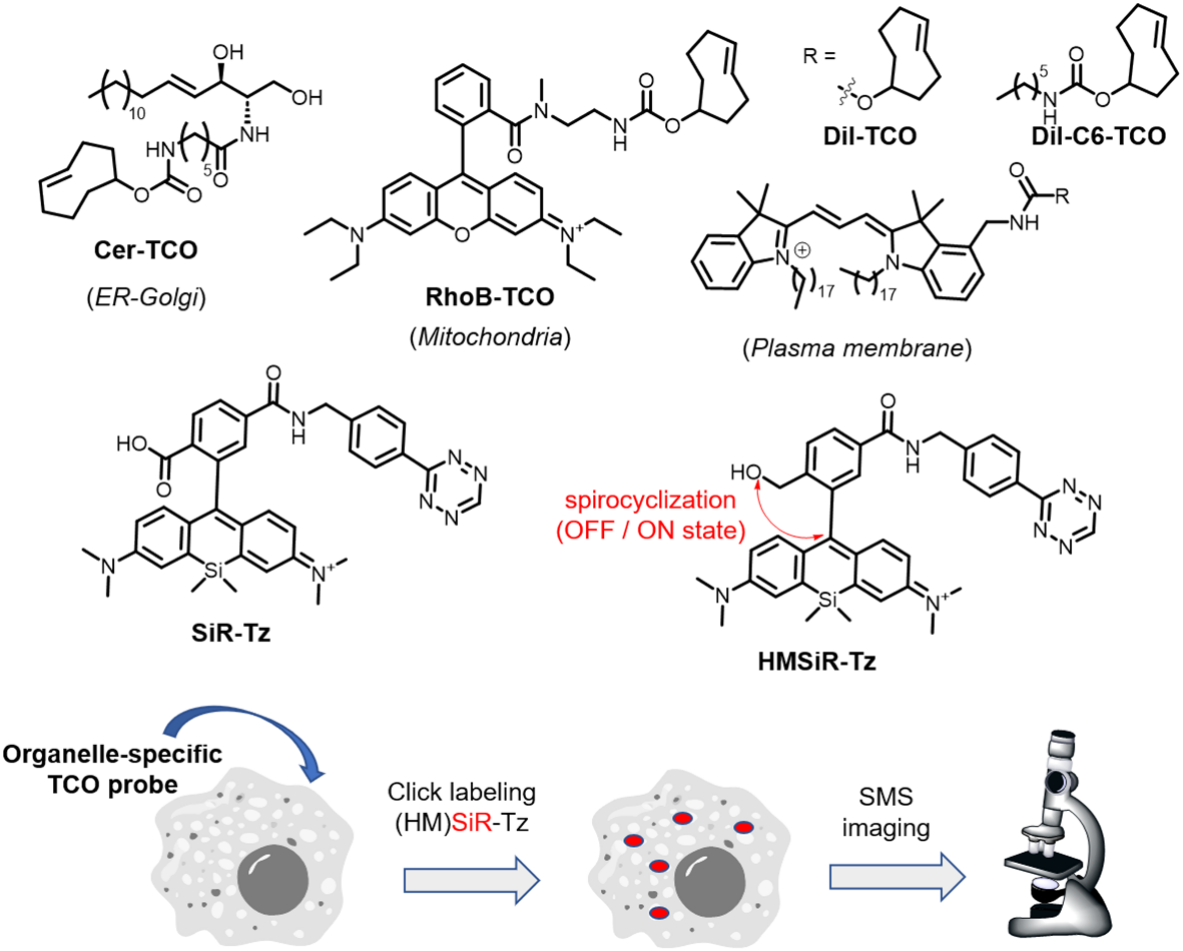
Structures of compounds facilitating intracellular assembly of HIDE probes for organelle-specific, long-term super-resolution microscopy

Super-resolution microscopy has revolutionized our ability to observe cellular structures and organelles [132]. However, developing suitable probes for this technique remains a formidable challenge. Ideally, these probes must achieve high-density labeling while employing exceptionally photostable fluorophores. Additionally, they must maintain compatibility with live cells to obtain information under biologically relevant conditions [133]. To address these challenges, Takakura et al. [134] developed near-infrared silicon rhodamines (SiR*–*Tz and HMSiR*–*Tz). These compounds were modified with a tetrazine moiety to bioorthogonally label cellular membranes containing preinstalled TCO groups (Fig. 29). For the HMSiR-Tz probe, spontaneous blinking occurs through a process called reversible intramolecular spirocyclization. This involves the probe transitioning between a closed, neutral dark state (OFF state) and an open, charged fluorescent state (ON state) [135]. Coupling these compounds with various membrane-localized probes containing TCO resulted in the acquisition of super-resolution movies lasting up to 30 min. These high-density environmentally sensitive (HIDE) probes were assembled in cells using click reactions. To achieve selective localization within the organelles, the authors employed ceramide (Cer), which targets both the Golgi and the ER, where mainly the Golgi are visualized at 20 °C and both organelles at 37 °C [136]. Additionally, they used a rhodamine B derivative for mitochondria staining and a dialkylindocarbocyanine (DiI) for plasma membrane visualization (PM). The movies acquired revealed the 2D dynamics of the mitochondria, plasma membrane, and filopodia as well as both 2D and 3D movements of the ER within living cells. Furthermore, the addition of the photoconvertible fluorescent protein mEos3.2 enabled two-color live-cell imaging.

**Fig. 30:**
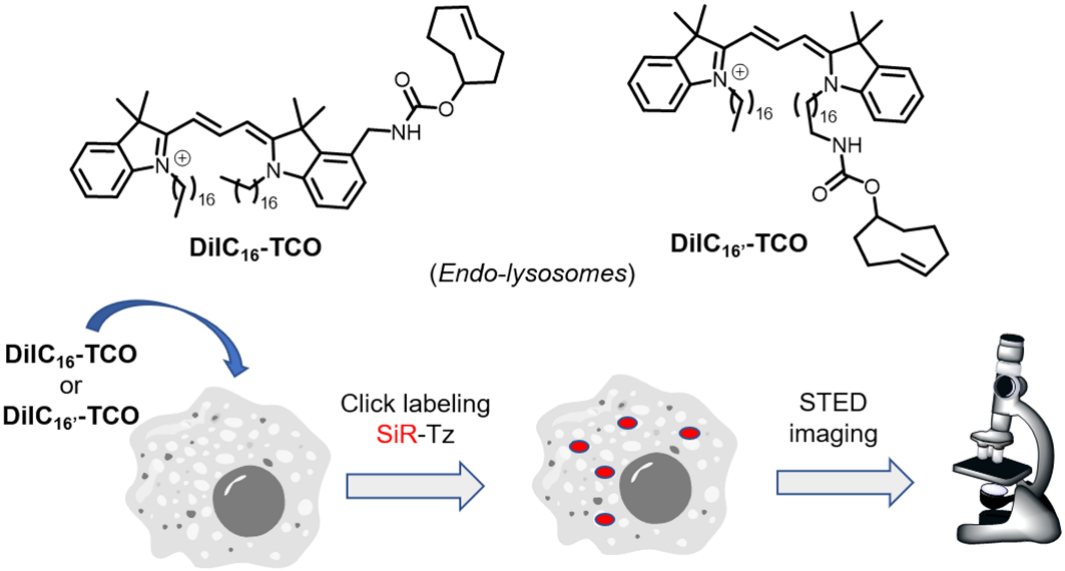
HIDE probes enabling visualization of endolysosomal membranes

In a subsequent investigation, the same research group developed the novel HIDE probes, DiIC_16_–SiR and DiIC_16′_–SiR, designed to tag and visualize endolysosomal membranes (Fig. 30) [137]. The probes were generated directly by reacting DiIC_16_–TCO and DiIC_16′_–TCO with SiR–Tz in live cells. Using stimulated emission depletion (STED) microscopy, the group successfully characterized endosomal dynamics in a set of genetically distinct fibroblasts derived from patients with mutations in the lysosomal transmembrane protein NPC1. The findings revealed previously unrecognized connections between endosome motility and specific mutations associated with Niemann-Pick type C disease. Significantly, these connections became discernible only through STED imaging.

### Visualizing Enzymatic Activity

**Fig. 31:**
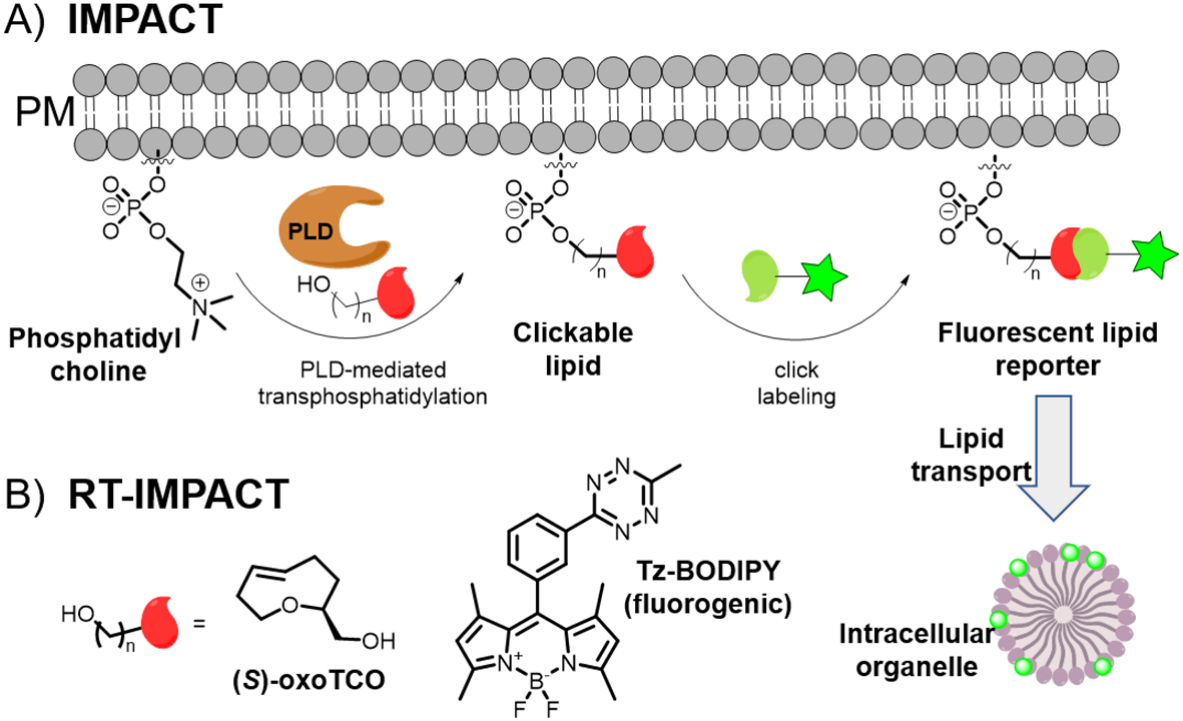
Metabolic labeling of phosphatidyl choline-containing membranes by transphosphatidylation using alcohols containing bioorthogonal TCO groups followed by fluorogenic IEDDA

Phosphatidic acid (PA) plays an important dual role as a key intermediate in phospholipid biosynthesis and a lipid second messenger responsible for influencing downstream protein functions [138]. The production of PA by enzymes such as phospholipase D (PLD) is essential in cellular processes: elevated PLD levels are linked to certain disease states, making PLD an important therapeutic target [139]. To characterize the subcellular activity of PLD, Baskin and colleagues developed an elegant strategy called imaging phospholipase D activity with clickable alcohols via transphosphatidylation (IMPACT). In this method, bioorthogonal functional groups are transformed into modified phosphatidyl lipids to enable click labeling with fluorophore probes [140]. Initially, the authors used alkynyl or azido alcohols for this purpose, demonstrating lipid labeling using CuAAC and SPAAC reactions [141, 142]. However, this technique was limited in its ability to precisely localize active PLD because of lipid diffusion and trafficking rates. To optimize the process, the authors developed a real-time version of the method, RT-IMPACT, based on rapid IEDDA reaction kinetics using tetrazines (Tz) and strained TCOs (Fig. 31).

Notably, even bulky TCO–alcohols proved efficient substrates for PLD enzymes, enabling their metabolic processing and incorporation into lipids. To identify the most suitable TCO for cellular experiments, screening was performed on a panel of TCOs with varying levels of hydrophobicity. The more polar oxo-TCO–alcohol [143], featuring an endocyclic ether group, was deemed the most appropriate choice, mainly due to its low background labeling signal. Interestingly, the (*S*)-oxoTCO alcohol outperformed the (*R*)-isomer. The optimized protocol facilitated real-time monitoring of PLD activity within seconds, enabling the observation of lipid translocation from the plasma membrane to the ER. Intriguingly, the internalization of these phosphatidyl alcohol lipids occurred through vesicle-independent pathways rather than conventional endocytic routes. This study underlines the potency of modern bioorthogonal reactions, especially those with fast kinetics and fluorogenic capabilities, to elucidate complex biological processes occurring in live cells on rapid timescales.

### Visualizing Cellular Membrane Dynamics

**Fig. 32:**
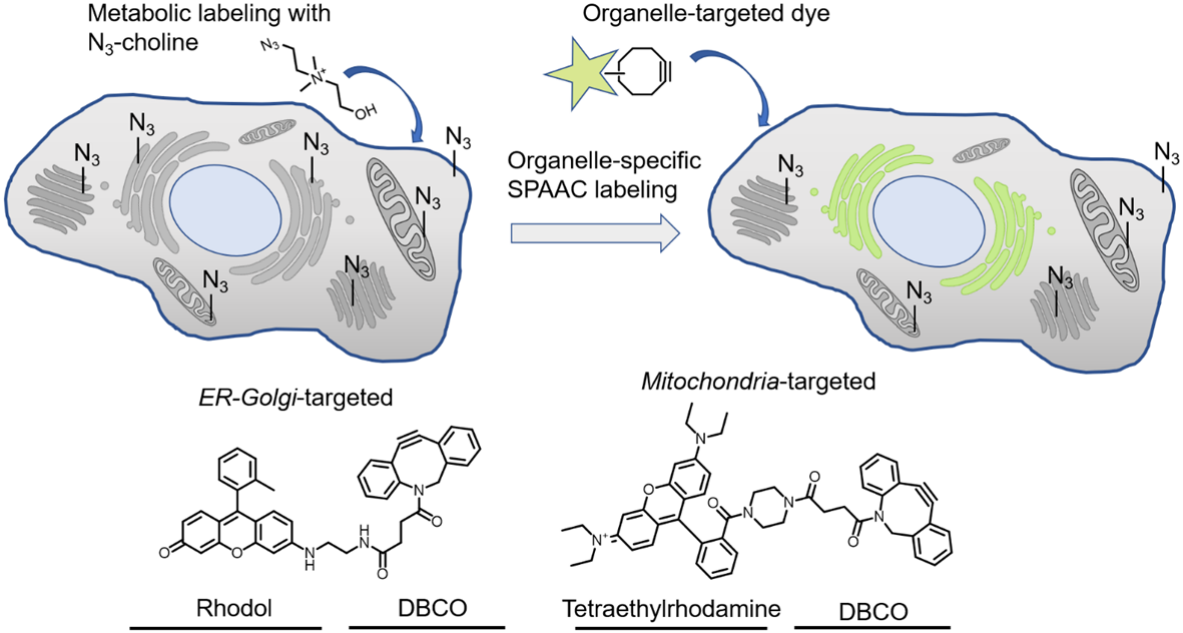
Organelle-specific SPAAC labeling after metabolic incorporation of azidocholine

Seeking a method to label and trace organelle membranes, Hamachi and co-workers combined metabolic labeling with an SPAAC reaction for the fluorescence imaging of organelles (Fig. 32) [144]. The method is based on the metabolic integration of azido-choline, which is processed by cells and incorporated into choline-containing phospholipids [145]. For precise spatial labeling, the researchers engineered targeted organelle probes. A rhodol–DBCO conjugate was used to achieve localization within the region occupied by the ER and Golgi. In this case, rhodol serves as the targeting element and DBCO as the reactive partner in the SPAAC reaction. In parallel, a tetraethylrhodamine DBCO conjugate was used to specifically target mitochondria. Interestingly, the intracellular concentration of rhodol–DBCO, used at a modest concentration of 100 nM, was remarkably elevated, reaching approximately 0.57 mM within the ER/Golgi region. This dramatic increase in concentration allowed for rapid labeling of N_3_-choline-modified lipids. Colocalization experiments confirmed the facility of the method in selectively labeling ER and Golgi membranes. Similarly, the rhodamine-based probe exhibited precise staining of live-cell mitochondria membranes. Owing to the distinct spectral properties of the two probes, each featuring a different fluorophore, both organelles were successfully imaged simultaneously.

After selectively labeling the membranes of distinct organelles, the authors then monitored the movement of labeled lipids between them. These investigations showed that phosphatidylcholine-containing lipids swiftly transitioned from their starting location in the ER/Golgi to mitochondria, the plasma membrane, and lysosomes within a few hours. In contrast, lipids originating in the mitochondria moved significantly more slowly, taking > 20 h to reach lysosomes. This technique also confirmed the role of the ER in supplying membrane lipids for autophagosome generation induced by starvation.

### Discovering Novel Genes Using Organelle-Targeted Reactions

**Fig. 33:**
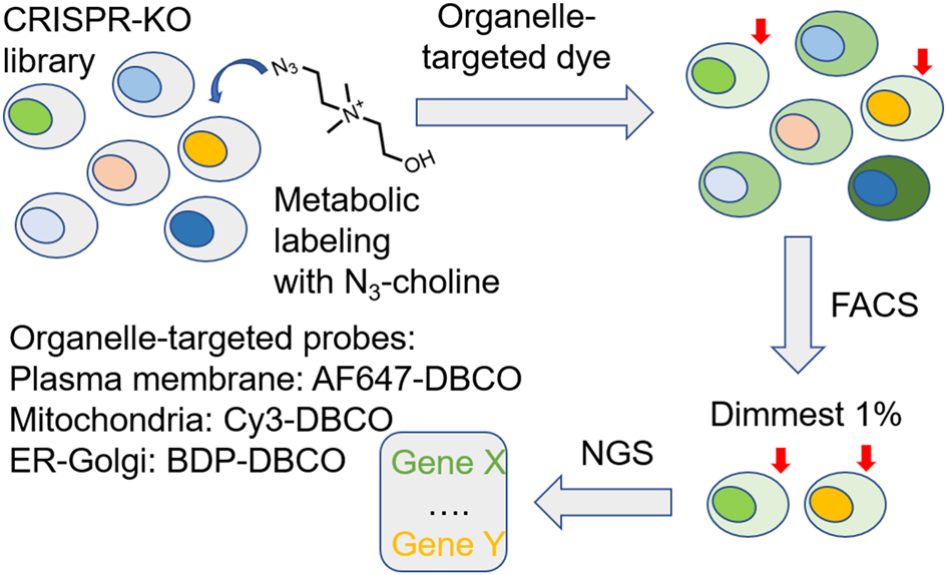
Identification of genes involved in phosphatidylcholine biosynthesis using a SPAAC reaction

In a follow-up study, the same group introduced a concept for selectively labeling organelles within cells called O-ClickFC, which stands for organelle-selective click chemistry coupled with flow cytometry. In this approach, cells are selectively labeled using metabolic treatment with azido-choline, fluorescence-activated cell sorting (FACS), and genome-wide clustered regularly interspaced short palindromic repeats gene knock-out (CRISPR-KO) screening [12]. The method involves the metabolic integration of azido choline into Cas9-expressing K562 cells, transduced with a genome-wide lentiviral single-guide RNA (sgRNA) library. Subsequent organelle-specific click-based staining, FACS sorting of cells exhibiting low fluorescence, and next-generation sequencing are used to generate a list of potential genes interfering with phosphatidylcholine biosynthesis. In this work, the authors evaluated a panel of DBCO dyes, including the BODIPY (BDP) DBCO conjugate for ER/Golgi staining, cyanine 3 (Cy3) for mitochondria, and cell-impermeable Alexa Fluor dyes (AF405, AF488, AF647) for labeling the outer plasma membrane leaflet (Fig. 33). The method was successful in identifying both established and novel human genes implicated in phosphatidylcholine biosynthesis at the subcellular level.

### Transition Metal Catalysis in the Plasma Membrane

**Fig. 34:**
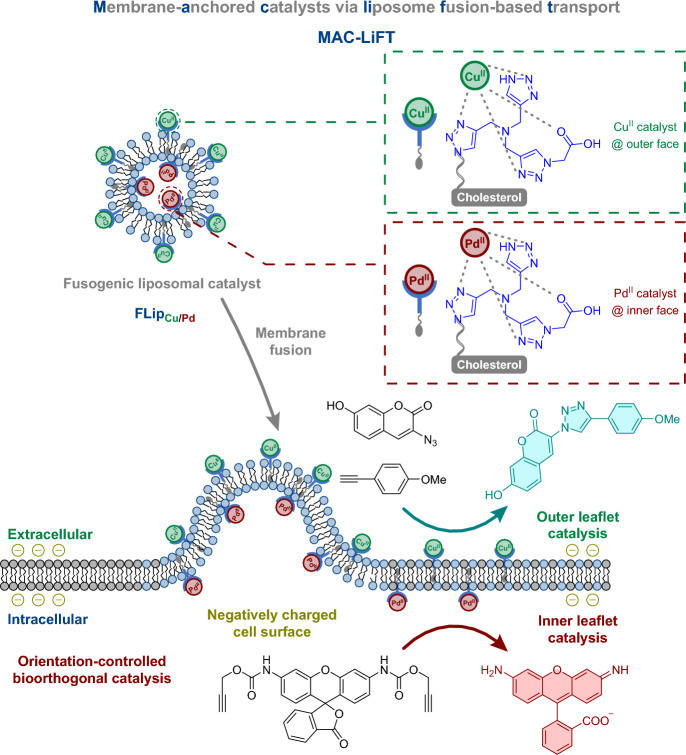
MAC-LiFT strategy with two types of bioorthogonal metal catalysts

The attachment of artificial catalytic systems to cell surfaces can provide cells with new synthetic functions. For this purpose, Chen et al. devised a strategy called membrane-anchored catalysts via liposome fusion-based transport (MAC-LiFT) [146]. They first developed catalyst-loaded fusogenic liposomes (FLip-Cat) (Fig. 34) containing Cu^II^ and/or Pd^II^ metal catalysts, which were then immobilized onto the liposomal membrane using a tris(triazolyl)amine (TTA) chelator linked to cholesterol through a poly(ethylene glycol) (PEG) linker. Additionally, fluorescein was attached to the TTA for cellular visualization. Two versions of the liposomal complexes were investigated: one with the catalyst on the outer side of the membrane (FLip_Cu/−−_) and the other with the catalyst on both the outer and inner faces (FLip_Cu/Cu_). Concurrently, FLip catalysts incorporating Pd(II) were also synthesized and tested.

FLip_Cu/Cu_ and FLip_Cu/−−_ were used in CuAAC reactions between ethynylanisole and azidocoumarin, while FLip_Pd/Pd_ and FLip_Pd/−−_ were employed in deprotecting reactions of propargyloxy-caged rhodamine. These Pd- and Cu-bearing FLip catalysts were remarkably efficient in facilitating fluorogenic reactions within HeLa cells. However, in the presence of EDTA or FSB during cellular reactions, only minimal fluorescence was observed with FLip_Pd/−−_ or Flip_Cu/−−_, presumably because of metal complexation with components in the solvent or environment. The efficacy of Pd- and Cu-FLip systems was also examined in MCF-7 and HepG2 cell lines.

Additionally, the researchers engineered asymmetrical Flips containing dual catalysts (Pd^II^ and Cu^II^) with controlled orientation, enabling spatially controlled bioorthogonal fluorogenic reactions on the cellular membrane. The selective deactivation of the catalysts using EDTA resulted in the effective regulation of reaction sites on the cell membrane.

Finally, MAC-LiFT was used to synthesize a bulky chimeric kinase binder inside cancer cells from two smaller molecules that can naturally penetrate the cells. This intracellularly synthesized chimeric molecule showed enhanced cytotoxicity at a significantly lower inhibitory concentration, highlighting the potential of MAC-LiFT in therapeutic applications.

## Detection of Signaling Molecules in Cellular Organelles

### Monitoring NO in Targeted Organelles

**Fig. 35:**
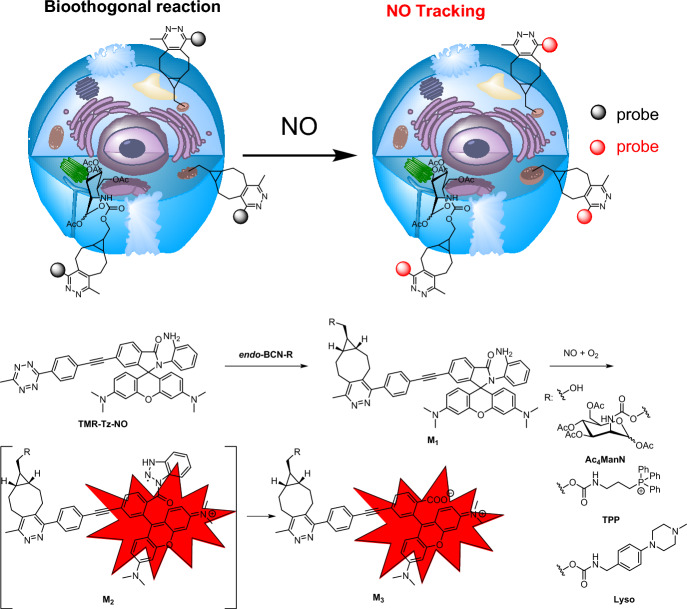
Bioorthogonal toolbox for monitoring NO in different organelles

Nitric oxide (NO) has essential biological roles as an unstable free radical, a messenger molecule, and a mediator in signal transduction. Given its regulatory influence on a wide range of physiological and pathological processes, detecting NO within living organisms is important [147]. However, because NO is unevenly distributed throughout cellular compartments, its accurate detection is a considerable challenge [148]. A common strategy for tracking NO involves conjugating a fluorophore to *o*-phenylenediamine, which functions as an NO scavenger for both exogenous and endogenous NO species [149]. To improve this tracking system, Xiao et al. introduced an NO-sensing approach based on a bioorthogonal IEDDA reaction [150]. This technique employs a NO-responsive TMR–Tz–NO probe, where tetramethylrhodamine-*o*-phenylenediamine is linked to a tetrazine moiety. In this probe, the fluorescence of the TMR fluorophore is quenched by the presence of the tetrazine group. This quenching can be reversed through an IEDDA reaction with a suitable dienophile such as BCN. To achieve targeted detection within specific organelles, the BCN scaffold was coupled to various targeting motifs, including triphenylphosphonium (TPP), morpholine, and acetylated mannosamine (Ac_4_ManN), enabling selective NO tracking within mitochondria, lysosomes, and membranes, respectively (Fig. 35).

TMR–Tz–NO effectively detected both exogenous and endogenous nitric oxide (NO) in living HeLa and Raw 264.7 cells. The authors also demonstrated successful tracking of NO in a live zebrafish inflammation model induced by lipopolysaccharide stimulation. This study highlights the potential of bioorthogonal reagents in targeting distinct cellular compartments in conjunction with analyte-responsive fluorophores for the sensitive detection of endogenous signaling molecules.

## Conclusions and Perspectives

In the field of bioorthogonal chemistry, organelle-targeted bioorthogonal reactions are uniquely tailored for investigating individual subcellular compartments. Researchers have developed and employed both metal-catalyzed and catalyst-free bioorthogonal reactions for the purpose of studying cellular organelles, which has enhanced our understanding of subcellular processes and cellular dynamics. Using innovative strategies, they continue to shed light on the specific roles of organelles in these processes, yielding important biological insights.

Approaches involving organelle-specific ligands and subcellular localization signals have been harnessed for accurate targeting. This precision has contributed to our exploration of organelle-specific interactions and functions, such as membrane potential dynamics, lipid translocation, antigen presentation, and real-time visualization of dynamic processes as well as enzymatic activities.

The use of bioorthogonal reactions in organelle targeting has the potential to be of immense therapeutic benefit. The development of organelle-specific probes is likely to result in discovery of novel therapeutic targets, innovations in personalized medicine, and more effective treatment strategies. These targeted approaches will not only enhance the efficacy of therapeutic agents but also mitigate concerns over off-target effects and resulting toxicity. Furthermore, integrating organelle-specific bioorthogonal reactions with emerging gene editing techniques increases the prospect of developing personalized therapies that address pathologies at the organelle level.

However, despite significant progress, several challenges remain. Innovative solutions are required to achieve complete organelle specificity, minimize off-target effects, and optimize delivery methods. The fast-paced nature of many cellular processes depends on imaging techniques with high temporal and spatial resolution. Therefore, continued advancements in super-resolution microscopy, single-molecule imaging, and real-time imaging techniques are essential for keeping pace with these developments. Developing probes with minimal cytotoxicity and high biocompatibility remains critical, as does the optimization of delivery mechanisms. Additionally, developing probes capable of targeting organelles and monitoring multiple cellular events simultaneously is needed to accurately monitor complex biological processes. Finally, progress in bioorthogonal chemistry, imaging technologies, and probe design goes hand in hand with the development of new advanced tools, increasing our ability to understand and analyze the biology of cells and organisms at the subcellular level.

In summary, organelle-targeted bioorthogonal reactions have advanced our knowledge of the complexities of cellular organization and function. As innovative methods continue to develop, our understanding of organelle biology and the range of techniques available for basic research and clinical applications are likely to increase further. Although challenges remain, the exciting prospects and potential breakthroughs in this field are set to reshape the landscape of cell biology and medical research in the years to come.

## Data Availability

The data reported in this review article are available on the internet, as indicated in the references below. The authors also confirm that the data and materials supporting the findings of this study are available within the article.
